# Borealisation of Plant Communities in the Arctic Is Driven by Boreal‐Tundra Species

**DOI:** 10.1111/ele.70209

**Published:** 2025-09-21

**Authors:** Mariana García Criado, Isabel C. Barrio, James D. M. Speed, Anne D. Bjorkman, Sarah C. Elmendorf, Isla H. Myers‐Smith, Rien Aerts, Juha M. Alatalo, Katlyn R. Betway‐May, Robert G. Björk, Mats P. Björkman, Daan Blok, Elisabeth J. Cooper, J. Hans C. Cornelissen, William A. Gould, Ragnhild Gya, Greg H. R. Henry, Luise Hermanutz, Robert D. Hollister, Annika K. Jägerbrand, Ingibjörg S. Jónsdóttir, Elina Kaarlejärvi, Olga Khitun, Simone I. Lang, Petr Macek, Jeremy L. May, Anders Michelsen, Signe Normand, Siri L. Olsen, Eric Post, Riikka Rinnan, Niels Martin Schmidt, Sofie Sjogersten, Anne Tolvanen, Joachim P. Töpper, Andrew Trant, Vigdis Vandvik, Tage Vowles

**Affiliations:** ^1^ School of GeoSciences University of Edinburgh Edinburgh UK; ^2^ CREAF Bellaterra (Cerdanyola del Vallès) Spain; ^3^ Faculty of Environmental and Forest Sciences Agricultural University of Iceland Reykjavík Iceland; ^4^ Department of Natural History Norwegian University of Science and Technology Trondheim Norway; ^5^ Department of Biology and Environmental Sciences University of Gothenburg Gothenburg Sweden; ^6^ Gothenburg Global Biodiversity Centre Gothenburg Sweden; ^7^ Institute of Arctic and Alpine Research (INSTAAR), University of Colorado Boulder Colorado USA; ^8^ Department of Ecology and Evolutionary Biology University of Colorado Boulder Colorado USA; ^9^ Department of Forest & Conservation Sciences, Faculty of Forestry University of British Columbia Vancouver British Columbia Canada; ^10^ Amsterdam Institute for Life and Environment, Vrije Universiteit Amsterdam Amsterdam the Netherlands; ^11^ Environmental Science Center, Qatar University Doha Qatar; ^12^ USDA Forest Service, Research and Development Río Piedras Puerto Rico USA; ^13^ Department of Earth Sciences University of Gothenburg Gothenburg Sweden; ^14^ Dutch Research Council Den Haag the Netherlands; ^15^ Department of Arctic and Marine Biology; Faculty of Biosciences, Fisheries and Economics UiT‐The Arctic University of Norway Tromsø Norway; ^16^ Department of Biological Sciences and Bjerknes Center for Climate Research University of Bergen Bergen Norway; ^17^ Department of Geography University of British Columbia Vancouver British Columbia Canada; ^18^ Department of Biology Memorial University St. John's Newfoundland Canada; ^19^ Biology Department Grand Valley State University Allendale Michigan USA; ^20^ Department of Electrical Engineering, Mathematics and Science, Faculty of Engineering and Sustainable Development University of Gävle Gävle Sweden; ^21^ Institute of Life and Environmental Sciences University of Iceland Reykjavík Iceland; ^22^ Organismal and Evolutionary Research Programme, Faculty of Biological and Environmental Sciences University of Helsinki Helsinki Finland; ^23^ Department of Arctic Biology University Centre in Svalbard Longyearbyen Norway; ^24^ Institute of Hydrobiology Biology Centre of the Czech Academy of Sciences Ceske Budejovice Czech Republic; ^25^ Department of Biodiversity and Nature Tourism Estonian University of Life Sciences Tartu Estonia; ^26^ Department of Biology and Environmental Sciences Marietta College Marietta Ohio USA; ^27^ Department of Biological Sciences Florida International University Miami Florida USA; ^28^ Department of Biology University of Copenhagen Copenhagen Denmark; ^29^ Department of Biology Aarhus University Aarhus C Denmark; ^30^ Faculty of Environmental Sciences and Natural Resource Management Norwegian University of Life Sciences Ås Norway; ^31^ Norwegian Institute for Nature Research Oslo Norway; ^32^ Department of Wildlife, Fish, and Conservation Biology University of California, Davis Davis California USA; ^33^ Department of Ecoscience Aarhus University Roskilde Denmark; ^34^ Arctic Research Centre Aarhus C Denmark; ^35^ School of Biosciences University of Nottingham Nottingham UK; ^36^ Natural Resources Institute Finland Oulu Finland; ^37^ Norwegian Institute for Nature Research Bergen Norway; ^38^ School of Environment, Resources and Sustainability University of Waterloo Waterloo Ontario Canada; ^39^ Swedish Environmental Research Institute Gothenburg Sweden

**Keywords:** boreal forest, boreal‐tundra ecotone, climate change, plant borealisation, tundra, vascular plants

## Abstract

Following rapid climate change, tundra plant communities are experiencing extensive compositional shifts. A conservation concern is the potential encroachment of boreal species into the tundra (‘borealisation’). Tundra borealisation has been sporadically reported, but not systematically quantified. Here, we synthesised data from across 32 study areas, spanning 1137 plots and 287 vascular plant species, resurveyed between 1981 and 2023. We (i) quantified tundra borealisation as the colonisation and increase in abundance of Boreal and Boreal‐Tundra species, (ii) assessed biogeographical, climatic and local borealisation drivers and (iii) identified species contributing to borealisation and their associated traits. Half of the plots experienced borealisation, although borealisation rates were not different to random expectation. Borealisation was greater in Eurasia, closer to the treeline, at higher elevations, in warmer and wetter regions, where climate change was limited, and where initial boreal abundance was lower. Boreal coloniser species were generally short‐statured, and more often shrubs and graminoids. Boreal species colonised around three times less frequently than Boreal‐Tundra species. Hence, our findings indicate that tundra borealisation is mainly driven by the spread of already established boreal‐low Arctic tundra species. These plant community composition changes could have cascading impacts on land‐atmosphere interactions, trophic dynamics and Indigenous and local livelihoods.

## Graphical abstract

We found limited borealisation rates across the tundra biome. However, borealisation was greater in Eurasia, closer to the treeline, at higher elevations, in warmer and wetter regions, where climate change was limited and where initial boreal abundance was lower. Boreal species colonised tundra plots less often than Boreal‐Tundra species. Boreal coloniser species were short‐statured, and more likely to be shrubs and graminoids.

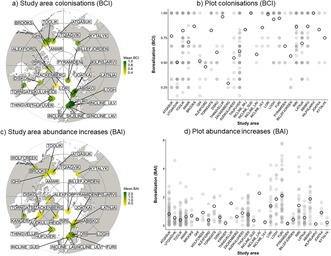

## 1 Introduction

Climate change is leading to widespread vegetation change in tundra regions (Elmendorf et al. 2012; García Criado et al. 2025; Myers‐Smith et al. 2011). Extensive changes to plant community composition have occurred, including changes in plant abundance and species range shifts (Elmendorf et al. 2012; García Criado et al. 2020) and treeline advance (Frost and Epstein 2014; Harsch et al. 2009; Rees et al. 2020). In this context, a conservation concern is that these shifts may include the encroachment of boreal species into the tundra (‘borealisation’; Speed et al. 2021). While borealisation has been extensively studied in the marine realm (Emblemsvåg et al. 2022; Pecuchet et al. 2020), terrestrial studies are lagging far behind (Verdonen et al. 2025). Within the tundra, range expansions of boreal animals such as red fox ( *Vulpes vulpes* ), moose ( *Alces alces* ) and songbirds have been reported (Elmhagen et al. 2017; Le Pogam et al. 2021; Tape et al. 2016), and recent modelling shows northward movement of boreal herbivores following warming temperatures (Speed et al. 2021). Despite some site‐specific studies in Russia, Alaska and Canada (Khitun et al. 2016; Roland et al. 2021; Timoney 2023), the extent to which plant borealisation (beyond treeline advance) is occurring at the tundra biome scale remains unquantified.

Borealisation is likely to be dependent on a combination of geographic, climatic and local factors. Sites closest to the tundra‐boreal forest ecotone might be more likely to experience successful plant dispersal and establishment from the boreal forest (Ropars and Boudreau 2012), especially those with no dispersal barriers (Rupp et al. 2001). Similarly, warmer conditions could favour boreal species as they inhabit a warmer thermal niche than tundra species (Lynn et al. 2021; Villén‐Peréz et al. 2020). This could result in regional differences in borealisation, since, for example, Arctic Europe (Palaearctic) is generally warmer than Arctic North America (Nearctic; Callaghan et al. 2004b). However, local conditions can influence the establishment of boreal species into the tundra (Dial et al. 2022), with herbivory potentially preventing plant colonisations (Eskelinen et al. 2017) and landscape structure driving adaptation and dispersal (Graae et al. 2018). Therefore, we could expect borealisation to be shaped by a combination of factors acting from macro to intermediate and local scales.

Certain species might contribute to borealisation more than others. For instance, species with large distributional ranges usually have a wider tolerance for environmental conditions (Lynn et al. 2021; Vincent et al. 2020) and are more likely to expand across tundra habitats (Callaghan et al. 2004a). Similarly, more competitive species such as shrubs and those with great dispersal potential and/or fast acquisition strategies (Aubin et al. 2016) could have greater geographic and climatic niches (Sporbert et al. 2021), and thus become more successful tundra colonisers. Hence, plant trait values such as tall height, high specific leaf area (SLA), low seed mass or low leaf nitrogen concentration might be linked to successful borealisation (García Criado et al. 2023; Vuorinen et al. 2017). Incoming species can re‐shape community traits, such as tundra plant communities becoming taller as a result of species turnover (Bjorkman et al. 2018a). Boreal species are generally taller than tundra plants and might outcompete them through shading, higher litter production and enhanced nutrient uptake (Kaarlejärvi et al. 2017; Pajunen et al. 2011; Thomas et al. 2020). Thus, the trait composition of tundra plant communities is likely to shift as boreal species become more abundant.

An influx of boreal species into the tundra will inevitably lead to changes in community composition, resulting in the widening or displacement of the boreal forest–tundra ecotone, a reduction of the tundra biome, or alternatively the creation of novel communities (Macias‐Fauria et al. 2012). The boreal forest and the Arctic tundra are distinct in terms of ecosystem functioning and service provisioning, and a loss of extent of the tundra biome would have global consequences (Callaghan et al. 2002). For example, a northward advance of the forest–tundra ecotone has been projected to decrease carbon capture in tundra ecosystems due to vegetation change, which together with enhanced permafrost thaw, could amplify climate change (Zhang et al. 2013). A reduction in tundra extent could also result in decreased habitat availability for tundra herbivores (Speed et al. 2021), and cascading impacts for trophic chains (Fauchald et al. 2017; Mallory and Boyce 2018) and Indigenous and local communities (Hupp et al. 2015; Rees et al. 2008).

Here, we synthesised observations of plant composition from across the Arctic to address the following research questions (RQs):

RQ1. How much borealisation has occurred across the tundra biome over the past four decades?

We expect that the majority of sites will have experienced an increase in boreal species presence and abundance, based on evidence from a few intensively studied sites to date (Khitun et al. 2016; Roland et al. 2021; Timoney 2023).

RQ2. What are the characteristics of sites that have experienced the most pronounced borealisation?

We hypothesise that borealisation has been greater closer to the treeline, at warmer sites, and where summer warming has been more pronounced, with thermal niches becoming available for warm‐adapted species (Elmendorf et al. 2015; Steinbauer et al. 2018). We expect the Palaearctic to experience greater borealisation relative to other Arctic regions given higher overall growing season temperatures and closer proximity of boreal ecosystems (Callaghan et al. 2004b).

RQ3. Which species are more likely to drive the borealisation of tundra plant communities and what are their traits?

We expect ubiquitous species to have expanded and colonised more often due to their wider tolerance and niche (Callaghan et al. 2004a; Lynn et al. 2021). Certain functional groups like shrubs and species such as mountain birch ( *Betula pubescens* ) might have greater expansion and colonisation rates due to their high dispersal capacity (Behrend et al. 2024; Myers‐Smith et al. 2011). We hypothesise that certain species traits associated with an acquisitive life strategy (e.g., high SLA), better competitive ability (e.g., tall stature), greater dispersal capability (e.g., lighter seeds) and lower palatability for herbivores (e.g., low leaf nitrogen; Aubin et al. 2016; Sporbert et al. 2021) will be associated with borealisation.

## 2 Methods

### 2.1 Plant Composition Data

We synthesised observations of plant composition from the database of the International Tundra Experiment (ITEX+; Henry and Molau 1997), a geographically distributed experiment with a pan‐Arctic extent and standardised study design and data collection (Molau and Mølgaard 1996). ITEX+ has a hierarchical structure, where each study area can contain several subsites, consisting of multiple sampling plots. We selected ITEX+ study areas in the Northern Hemisphere included within the boreal forest and tundra biomes according to the WWF ecoregions (Olson et al. 2001), which included Oro‐Arctic (near‐Arctic alpine sites), sub‐Arctic and Arctic sites, which were then categorised as Arctic (*n* = 642 plots within 71 subsites and 15 sites) or alpine (*n* = 495 plots within 42 subsites and 17 sites) for analysis. We used only permanently marked control plots (i.e., with no experimental treatments) that had been surveyed at least twice over a minimum of five years, as shorter time series can overestimate trends in tundra plant communities due to interannual variability (Harris et al. 2022; Valdez et al. 2023). Our dataset consisted of 33388 records from 1137 plots within 113 subsites in 32 study areas surveyed at least twice between 1981 and 2023, encompassing 287 vascular plant species. There was an average of 10 plots per subsite (range = 1–83), 3.5 subsites per study area (range = 1–31) and 15 years between samplings (range = 5–28). Plots were our replication unit for analyses, with an average size of 3.86 m^2^ (range = 0.05–120).

We followed the protocol published in García Criado et al. (2025) to convert vascular plant abundance values to relative cover (0%–100%) within each plot in order to ensure consistency across survey methods (i.e., point‐intercept data and cover‐equivalent data). Thus, total cover in each plot by year always added up to 100%. We retained only vascular plants (since non‐vascular plants are not recorded consistently across the ITEX+ dataset) and removed abiotic records (e.g., rocks, soil, etc.) prior to cover calculations. In total, 37,031 abiotic and non‐vascular plant records were removed. To standardise survey effort across sites, we removed plot by year combinations that reported > 10% morphospecies (i.e., plants not identified to species level). We standardised the taxonomy of all species within the dataset following WorldFlora Online as of October 2024 (WFO 2024). Specifically, we matched species names in our dataset with those in WFO via the ‘WorldFlora’ R package (Kindt 2020) and aggregated subspecies and varieties to species. Since the presence of ecotypes and an evolving taxonomy is a well‐known issue for tundra plants, we assigned the latest accepted synonym from WorldFlora Online for consistency among species.

### 2.2 Species Classification

While there are different methods to categorise species according to their native distributions, we followed a standardised approach for consistency across species and regions by using the Arctic Biodiversity Assessment (Meltofte 2013) to classify vascular plant species. In particular, we followed Appendix 9.1 (Daniëls et al. 2013), which lists all Arctic vascular plant species and their distribution in the five Arctic subzones based on Elven (2007). We created groupings with all possible combinations of distribution and frequency across the Arctic and boreal zones (see Table S1 for a detailed classification). We considered a species to be present in a particular zone when it was reported as ‘scattered’ or ‘frequent’ in Daniëls et al. (2013); in contrast, we considered a species to be absent in a particular zone if it was described as ‘rare’, ‘uncertain’ or ‘introduced’ (Table S1). When several subspecies or varieties per species were listed in Daniëls et al. (2013), we standardised them at the species level by assigning the more abundant category for each zone. For the nine species in our dataset that were not included in Daniëls et al. (2013), we checked their distribution in the Global Biodiversity Information Facility (GBIF 2024) to manually assign them a class. Carrying out random checks of the Daniëls et al. (2013) classification against GBIF ranges resulted in comparable species classifications (not shown). Overall, we defined four classes based on species' geographical ranges across the boreal and tundra biomes (Elven 2007; Walker et al. 2005):

- *Boreal*: species found in the boreal but not in the tundra biome, *n* = 16.
- *Boreal‐Tundra*: species distributed in the boreal zone and Low Arctic tundra (subzones D and E), but not the High Arctic tundra (subzones A–C), *n* = 150.
- *Arctic*: species found in the tundra biome but not in the boreal zone, *n* = 14.
- *Ubiquitous*: species found in the boreal zone, Low Arctic and High Arctic, *n* = 107.

These classes are relative to these two biomes and are not intended to reflect the full range of species distributions in other biomes (e.g., a ‘Boreal’ species could also extend into the temperate forest). While the number of species included in each class is relatively unbalanced, the species classification reflects the ecological nature of the species and the distribution of the ITEX+ network within the tundra biome. Hereafter, we capitalise the word ‘Boreal’ when referring to the specific species class, and consider ‘boreal’ (in lowercase) to include species distributed across the biome boundary but not extending into the High Arctic (i.e., Boreal and Boreal‐Tundra species classes). We consider Boreal‐Tundra species to contribute to borealisation on the basis of their evolutionary history. In most cases, current species distributions in the boreal and Low Arctic zones are the result of expansions from boreal into Arctic locations that were previously glaciated (Aarnes et al. 2012; Birks 2008), underscoring the importance of warmer periods following deglaciation. Thus, expansions of these species represent long‐term borealisation processes. Boreal‐Tundra species could also include those that survived glaciations in refugia (Abbott et al. 2000), but these are very likely a minority compared to the former (Alsos et al. 2022).

### 2.3 Borealisation Indices

We assessed borealisation in each plot (i.e., at the community level) both in terms of colonisations and abundance changes of boreal species, using two complementary indices to better untangle these two processes of plant community change. First, we defined the Borealisation Colonisation Index (BCI; RQ1). This metric considers the start and end time points of monitoring per plot and reflects the influx of new boreal species into a plot over time. BCI reflects the proportion of colonisers that are boreal species (Boreal and Boreal‐Tundra species) and is bound between 0 and 1. Thus, a value of BCI = 1 does not mean that the plot is composed exclusively of boreal species; rather, that all the plot colonisations are from boreal species. Duration is not explicitly included in this formula because we were interested in the proportion of total colonisations that were boreal. Still, we found no relationship between BCI and duration (slope = 0.001, CI = −0.017 to 0.018; zero–one inflated beta distribution). BCI is calculated as follows:

$$\mathrm{BCI}=\frac{\text{Colonising boreal species}}{\text{Total colonising species}}$$

To quantify borealisation per plot based on changes in abundance of boreal species, we defined the Borealisation Abundance Index (BAI; RQ1). This metric reflects how ‘borealised’ a plot has become over time, considering the values of relative boreal cover (Boreal and Boreal‐Tundra species) at the start and end time points of monitoring per plot. BAI quantifies the rate of change in proportional cover of boreal species per year per plot. BAI is expressed as a rate (% cover change per year) and can be positive or negative. Because total cover in each plot by year always added up to 100%, any increase in boreal species abundance can be interpreted as a parallel decline of Arctic and Ubiquitous species and vice versa. BAI is calculated as follows, where *b* refers to boreal species:

$$\mathrm{BAI}=\frac{\left(\Sigma b\;\mathrm{End}\;\text{cover}-\;\Sigma b\;\text{Start cover}\right)}{\text{Duration}\;\left(\text{years}\right)}$$

We calculated both BCI and BAI as the difference between the end and start time points per plot rather than fitting linear models to calculate slopes for plots for which we had multiple observations over time. This is due to the fact that BCI is a colonisation index, thus the starting value would always be 0 (since there are no colonisers in the first time point). In practice, this means that the index could have a negative value due to fluctuations in the number of boreal colonisations over the years, and thus the end‐start method gives a more accurate representation of colonisations. Since BAI had a strong correlation between the end‐start method and linear models (Pearson's *r* = 0.97, *p* < 0.001), we opted for consistency by calculating both BCI and BAI in a similar manner.

In order to understand whether BCI and BAI reflected actual borealisation dynamics or background community turnover, we contrasted our observed index values with a null model. The BCI null model was built by resampling 999 times the species classes for all the species present in each study area over the study period and subsequently calculating mean BCI per plot from this randomisation, together with the interval encompassing ±2 SD. For BAI, we similarly randomised 999 times the species classes and calculated cover change as the difference between end and start cover for the simulated boreal species. To assess similarity between observed and simulated values, we compared the observed values to the simulated mean BCI and BAI ±2 SD per plot.

### 2.4 Drivers of Community‐Level Borealisation

We selected relevant drivers of community‐level borealisation reflecting main biogeographical (latitude, biome, distance to treeline, barriers to dispersal and biogeographic region), climatic (climatologies of summer temperatures, minimum annual temperature and annual precipitation and their associated climate change) and local factors (herbivory intensity, dominant grazer, elevation, moisture, plot size, permafrost and initial number or abundance of boreal species).

For each subsite (i.e., the level at which geographic coordinates were available), we extracted latitude and biome (Oro‐Arctic or Arctic) information, as defined in Olson et al. (2001). Additionally, we calculated the distance to the nearest treeline. For Arctic plots (those north of the latitudinal treeline), we calculated the distance to the latitudinal treeline from the Circumpolar Arctic Vegetation Map (Raynolds et al. 2019) on QGIS (version 3.30.2), and for Oro‐Arctic plots, we calculated the distance to the elevational forest line using satellite data on Google Earth (version 10.65.1.2). For Oro‐Arctic plots, we corrected the distance to the elevational treeline to reflect the ‘ground distance’ by calculating the hypotenuse, considering the elevation of the subsite and of the elevational forest line. Distance to treeline was centred on zero by subtracting the mean to allow for model convergence. We categorised barriers to dispersal based on the type of topographical features that were found between the subsite and the latitudinal (for Arctic plots) or elevational treeline (for Oro‐Arctic plots) as: uninterrupted, small water bodies (e.g., lakes, rivers), mountains and large water bodies (e.g., seas, oceans). We also categorised each plot by its biogeographic region, according to glaciation history (Ray and Adams 2001) into Eastern North America, Western North America, Greenland‐Iceland and Eurasia.

We extracted climatic data from CHELSA v2.1 for each subsite for the time period 1980–2019 at a resolution of 1 × 1 km (Karger et al. 2017). We calculated both climatologies (average value per climatic variable over time) and change over time (as slopes of climate variables over the years) for the following climatic variables: summer temperature (mean of the June, July and August months, to reflect growing season conditions), minimum annual temperature (as the mean daily minimum air temperature, to reflect plants' capacity to withstand cold) and annual precipitation (to reflect comparative water availability). We removed climatic data for 1980–1983 mean annual temperature and for 2005 minimum temperature since the files contained obvious data errors. The value of precipitation change of 18.24 mm per year at the INCLINE_SKJ subsite was removed as it was a clear outlier. This high value is likely due to the quantification of climatic values in extremely rugged terrains including fjords and mountains. Despite this outlier, CHELSA remains the most appropriate choice for our climate data due to its very fine resolution at 1 × 1 km and because its quasi‐mechanistical statistical downscaling has outperformed other interpolation‐based climatic data sources, particularly for precipitation (Karger et al. 2017).

Finally, we included variables at the subsite level available in the ITEX+ dataset, as provided by the site principal investigators, relating to biotic interactions (herbivory intensity, dominant grazer), local environmental conditions (elevation, moisture) and sampling effort (plot size). Herbivory intensity was categorised as low, medium and high. Dominant grazer included none, insects, birds, small mammals and large mammals. Moisture was also a categorical variable: dry, moist, wet and mixed. We extracted permafrost data for each subsite from Obu et al. (2019) as the Permafrost Probability Function (100 m pixel size), which was then converted into standardised categories (none, sporadic, discontinuous, continuous). Initial boreal status was calculated at the plot level as the number of Boreal and Boreal‐Tundra species present at the start of the plot monitoring period for BCI models, and as the total relative cover of Boreal and Boreal‐Tundra species at the start of the monitoring period for BAI models.

### 2.5 Species Trait Data

We extracted plant trait data from TRY v6.0 (Kattge et al. 2020), which includes trait values from multiple campaigns, including the Tundra Trait Team (Bjorkman et al. 2018b). For each species, we extracted georeferenced records found north of 50° latitude (the southernmost limit of the boreal forest) for plant height (m), specific leaf area (SLA; mm^2^/mg), leaf nitrogen (mg/g), leaf C:N (g/g) and seed mass (mg). We also kept non‐georeferenced trait data when it was evident from metadata that the records had been taken at locations north of 50° latitude. We retained data for those species that had a minimum of five records per trait. We removed 118 outlier records (i.e., the value was greater than 5 SD of the mean trait value per species). The final dataset contained trait data for 191 species (plant height), 166 species (SLA), 83 species (seed mass), 120 species (leaf N) and 54 species (leaf C:N). We calculated the mean trait values for each species, which were then incorporated as fixed effects in the species‐level models (see below). Additionally, we included the following categorical traits: woodiness (woody, not woody), deciduousness (evergreen, deciduous), N‐fixing capacity by symbiosis with N_2_‐fixing bacteria (fixer, non‐fixer), berry production (berry producing, non‐berry producing), taxonomic family (including 36 families) and functional group (shrubs, forbs, graminoids). While certain shrubs in our dataset could potentially reach enough height to become a tree, particularly outside of the Arctic, we do not make a distinction between these and refer to them as shrubs.

### 2.6 Community‐Level Models

To assess the drivers of community borealisation, both for BCI and BAI, we fitted three Bayesian hierarchical multivariate models that reflected the effect of the different variables at different scales (RQ2; Table S2): (1) biogeographical model, (2) climatic model and (3) local model. We also tried fitting a single model including all predictor variables of interest, but this model failed to converge.

To characterise plots with boreal expansions only (Tables S2 and S3), we retained those plots whose average BCI or BAI was > 0; hereafter ‘positive‐only models’. The plots included in each dataset differ (BCI: *n* = 598 plots, BAI: *n* = 488 plots) because plots that had experienced boreal colonisations did not necessarily undergo increases in abundance of boreal species on average and vice versa. To characterise the full gradient of change (Tables S2 and S3), we also ran models with the same fixed effects including plots without boreal colonisers (i.e., plots with 0 values for BCI, *n* = 1137) or plots with no change or reductions in abundance of boreal species (i.e., plots with negative and 0 values for BAI; *n* = 1137), hereafter ‘full‐range models’. When plots never had boreal species at the start or at the end, or they had boreal species in between timepoints, but not at the start and/or end survey timepoints, they were assigned a BCI and/or BAI = 0 accordingly. We interpret the BCI ‘positive‐only’ models to indicate the ‘borealness’ of the colonisers, while the ‘full range’ BCI models inform about both the likelihood of borealisation occurring, and the ‘borealness’ of those colonisations. BAI ‘positive only’ models refer to the boreal abundance increases, while BAI ‘full‐range’ models reflect the ‘net borealisation’ of the community. Finally, to assess how well colonisations by boreal species represent net borealisation of the plots (i.e., an increase in the representation of boreal species between the two timepoints), we calculated the net change per plot as the difference between boreal colonisations and boreal losses. We found that BCI and the net change values are positively correlated (Figure S1a), indicating that a high BCI value generally corresponds to a net increase in the number of boreal species in a plot.

Prior to building the models, we assessed pairwise correlations among all the potential predictor variables for each model type (biogeographic, climatic and local) within the ‘positive‐only’ dataset. Only complete pairwise observations were included, i.e., those plots that had data available for all predictor variables. We calculated Spearman's rank correlation indices between pairs of variables for the subsets of variables included in each of the three community‐level models (Figure S2). Given that pairwise correlations can only be computed for continuous or ordinal data, we coded categorical variables as ordinal values (see Figure S2). For strongly correlated variables (absolute Spearman's rank coefficient > 0.7), we kept the variables that more closely aligned with suspected mechanisms of change, as per our hypotheses. In the biogeographical models, we removed latitude and barriers to dispersal, as they were strongly correlated with distance to treeline and biome, which were our main hypotheses (Figure S2a). In the dataset for BAI, biome was also removed as it was strongly correlated with distance to treeline (Figure S2b). In the climatic models, we removed minimum temperature climatology, which was strongly correlated with precipitation climatology, and we only had one climatology variable related to precipitation but multiple related to temperature (Figure S2c,d). No predictor variables were removed from the local models (Figure S2e,f).

We employed a Bayesian framework for all analyses. All community models included subsite as a random effect to account for the ITEX nested sampling design (Tables S2 and S3). Models had different error distributions depending on the structure of the response variable: Gaussian with an identity link function (for response metrics with a normal distribution, e.g., BAI models), beta with a logit link function (for values between 0.0001 and 0.9999; e.g., the BCI positive‐only models, subtracting a constant of 0.0001 for BCI to fit the data into a beta distribution) and binomial models with a logit link function for integer count values (e.g., the BCI full‐range models with values between 0 and 1). Binomial models are used to model proportions where the response variable is a count of cases that can fall into only one of two classes (Dunn and Smyth 2018). Here, we used the binomial family to model BCI per plot as the count of boreal colonisers out of the total number of colonisers.

### 2.7 Species‐Level Models

To assess which species and species classes are more likely to drive the borealisation of tundra plant communities (RQ3), we calculated how many times each species had colonised different plots (i.e., times colonised, expressed as number of plots), and its average abundance change per year across all plots. We then modelled times colonised (only those species that had colonised at least once) and average abundance change (only those that had increased in abundance across plots) as a function of species class. As above, we calculated the net change per species as the difference between the number of plots colonised and the number of plots where the species became extinct. We found a positive correlation between times colonised and net change per species (Figure S1b), indicating that more frequent colonisations per species generally correspond to net abundance increases.

To identify which traits are associated with species contributing to borealisation (Boreal and Boreal‐Tundra species), we retained those species that had colonised at least once (for colonisation models) and that had increased in abundance on average across plots (for abundance models). Then, we modelled times colonised and abundance change as a function of relevant plant traits (Tables S2 and S3). Continuous traits were log‐transformed as species differences are better characterised on a log scale (Bjorkman et al. 2018a; Westoby 1998). Similar to the community‐level models, we assessed pairwise correlations between traits using Spearman's rank correlation indices for all trait variables (Figure S3). Categorical traits were transformed to ordinal variables for investigating correlations (Figure S3). We removed Leaf C:N from the species models, as this trait is inherently correlated with Leaf N (Figure S3) and Leaf N had a greater number of available records. Leaf C:N was also correlated with SLA and seed mass in the colonisation dataset. To enable model convergence, we did not include the following categorical variables: woodiness (because it is exclusively associated with shrubs, one of the categories of the ‘functional group’ variable), berry production (as there were only nine berry‐producing shrubs), deciduousness (as there were only 27 deciduous shrubs and 16 evergreen shrubs, while this category was not applicable to the remaining 228 [83.8%] species), taxonomic family (since 11 [30.5%] family groups had just one species) and N‐fixing capacity (because there were only 10 N‐fixer species). Therefore, even though we originally extracted data for 11 traits, only five (plant height, SLA, seed mass, leaf N and functional group) were eventually included in the species models as a result of correlations between traits and uneven sampling sizes.

Since the trait model reduces sample size to those species with data for all traits (Table S2), we fitted additional univariate models to identify any potential differences when using a larger sample size. Species‐level models had a negative binomial data distribution with a log link function (for count data where the variance is greater than the mean, e.g., the ‘times colonised’ models) and a Gaussian distribution with an identity link function (for response metrics with a normal distribution, e.g., the ‘mean abundance change’ models). We specified weakly informative priors [as gamma (0.01, 0.01)] for the dispersion parameter of the negative binomial family.

To better understand the relationship between species‐level gains and losses, we calculated the number of times that a species was lost from a plot (‘times lost’) and modelled its relationship with times colonised. Finally, to understand whether these traits were exclusively associated with colonisations or they reflected turnover dynamics through a greater number of extinctions, we also modelled ‘times lost’ as a function of the different traits.

### 2.8 Software

We used the software and programming language R version 4.2.0 (R Core Team 2022). Bayesian models were fitted using the ‘brms’ package (Bürkner 2017). We ran four chains per model, each chain with 2000 iterations, and 400 iterations of warm‐up, with an ‘adapt_delta’ value of 0.8 (the average probability of accepting a posterior draw) and ‘max_treedepth’ of 10 (depth of the tree being evaluated in each iteration). Convergence was assessed through examination of the *R*_hat_ term and trace plots. Correlation plots were visualised with the ‘corrplot’ package (Wei and Simko 2021). In all Bayesian models, we considered an indication of statistically clear effects if the 95% credible intervals did not overlap zero.

## 3 Results

### 3.1 Community Analyses

**FIGURE 1:**
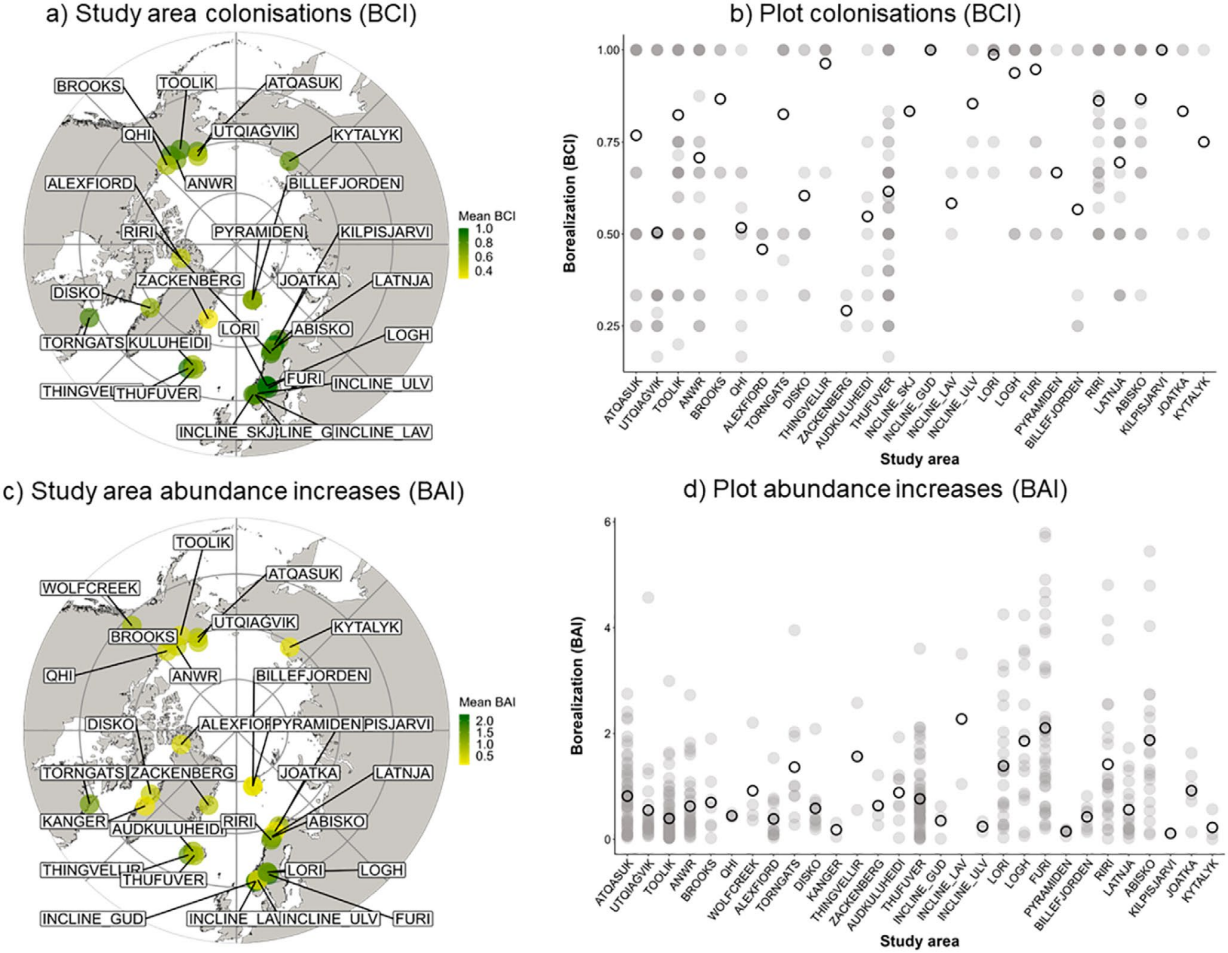
The magnitude of boreal plant community colonisations (BCI) and abundance increases (BAI) varied across the tundra. (a) BCI estimated as the average of the plots within a study area that experienced colonisations of boreal species (BCI > 0), (b) BCI index of those plots within each study area, (c) BAI estimated as the average of the plots within a study area that experienced an increase in the abundance of boreal species (BAI > 0), (d) BAI index for those plots within each study area. Points in (a) and (c) are coloured according to the magnitude of increase (as BAI and BCI) as a study area average. Open circles in (b) and (d) indicate the mean value of the plot borealisation index at the study area level, which represent the same value as coloured points in (a) and (c). Study areas in (b) and (d) are arranged by longitude. Darker grey colours indicate overlap of multiple points. Note that this figure shows the magnitude for plots that experienced increases in boreal species; for an analysis that includes plots where boreal increases did not occur (BCI = 0 and BAI ≤ 0), see Figure S4.

Boreal species colonised 52.6% of plots, with strong variability in magnitude across plots (Figure 1a,b). When considering only plots that had experienced boreal colonisations (i.e., BCI > 0, *n* = 598), mean BCI across plots was 0.77 (range = 0.16 to 1). When including zero values (*n* = 1137), mean BCI was 0.4 (range = 0 to 1; Figure S4a,b). Similarly, boreal species increased in abundance at 42.9% of plots, also with considerable variation across plots (Figure 1c,d). For plots with BAI > 0 (*n* = 488), mean BAI was 0.93% increase per year (range = 0.007 to 5.79). For the full range of values (i.e., ‘net borealisation’; *n* = 1137), mean BAI was −0.09% per year (range = −5.82 to 5.79%, CI = −0.2 to 0.1, Figure S4c,d). When comparing the observed BCI values with those expected by chance (i.e., null models), we found that observed values were only different from those simulated by chance (mean ± 2 SD) in 2.5% of plots. For BAI, observed values were different from those simulated in 7.4% of plots.

**FIGURE 2:**
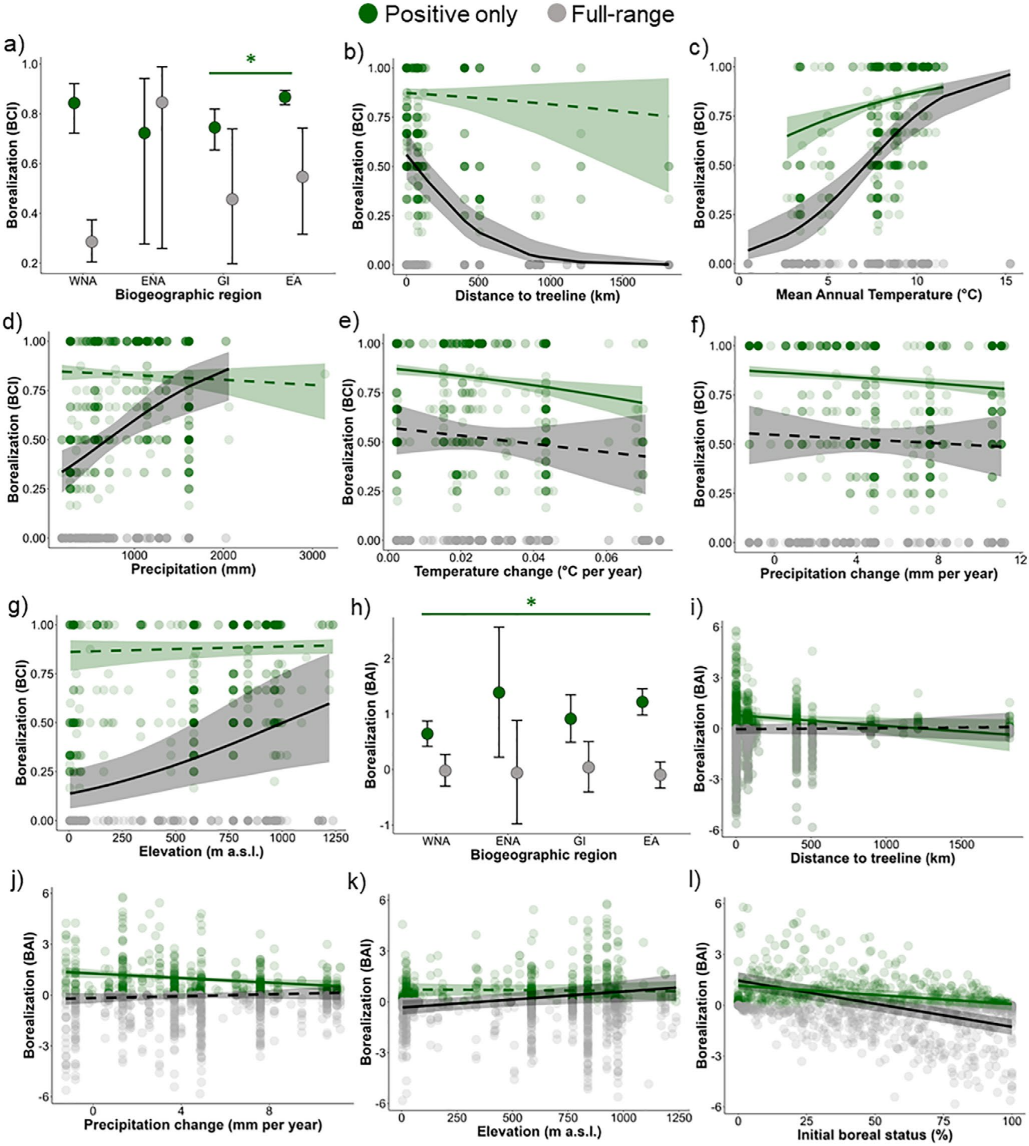
Borealisation was associated with biogeographic, climatic and local variables across the tundra biome. Borealisation was estimated using boreal colonisations (BCI; a–g) and abundance change (BAI; h–l). As assessed using multivariate models, colonizations by boreal species were more likely to occur (i.e., ‘full‐range models’) at sites closer to the treeline (b), warmer and wetter sites (c, d) and at higher elevations (g), while greater magnitudes of boreal abundance increases (i.e., ‘positive‐only models’) occurred at sites in Eurasia (EA) versus Greenland‐Iceland (GI), while other regions overlapped (ENA = Eastern North America, WNA = Western North America; a), and at warmer sites (c) with the least amount of climate change (e, f). Multivariate models show that increases in abundance of boreal species were more likely (i.e., ‘full‐range models’) at higher elevations (k) and in plots with lower initial boreal status (l). The magnitudes of abundance increases (i.e., ‘positive‐only models’) were stronger in Eurasia (EA) than in Western North America (WNA; h), at sites closer to treeline (i), that had experienced the least increases in precipitation (j) and that had lower initial boreal abundance (l). The panel shows all variables that were significant in at least one of the two multivariate models (i.e., ‘positive‐only’ and ‘full‐range’ models). Lines and semi‐transparent ribbons represent the model estimate and 95% credible intervals, respectively, and are coloured according to model type (i.e., ‘positive‐only’ in green and ‘full‐range’ in grey). Solid lines indicate 95% credible intervals of fixed effects that did not overlap zero and dashed lines indicate 95% credible intervals that overlapped zero. Each point represents a plot, with darker colours indicating point overlap. Green points are included in both the ‘positive‐only’ dataset (i.e., positive values only, BCI: 598 plots, BAI: 488 plots) and in the ‘full‐range’ dataset (i.e., including also zeroes and negative values, BCI and BAI: 1137 plots). Grey points indicate plots only included in the ‘full‐range’ dataset (i.e., zeroes and negative values), in addition to the positive values. Asterisks indicate when two categorical variables differed significantly from each other, coloured according to model type.

Within plots that had experienced boreal colonisations (i.e., ‘positive‐only’ BCI models), mean BCI was greater at sites in Eurasia (EA) and Western North America (WNA), and were more variable in Eastern North America (ENA). BCI was lower in Greenland‐Iceland (GI) than in EA (Figure 2a). BCI was also greater at sites that were relatively warm (Figure 2c), had warmed least over time (Figure 2e) and increased least in precipitation (Figure 2f), though the effect size of climate change was relatively small (Table S3). When considering the ‘full‐range’ BCI models (i.e., including zeroes), we found that boreal colonisers were more likely to occur closer to treeline (Figure 2b), in warmer and wetter sites (Figure 2c,d), at higher elevations (Figure 2g) and in larger plots (not shown; slope = 0.014, CI = 0.0051 to 0.0234).

The magnitudes of boreal abundance increases (i.e., ‘positive‐only’ BAI models) were relatively similar across regions, but they were stronger in EA compared to WNA (Figure 2h). BAI was greater at sites closer to treeline (Figure 2i), that had increased least in precipitation (Figure 2j) and that had lower initial boreal abundance (Figure 2l), though these three had relatively weak effect sizes (Table S3). In the ‘full‐range’ BAI models (i.e., including zeroes and negative values), net borealisation was greater at higher elevations (Figure 2i) and where the initial abundance of boreal species was lower (Figure 2l), though again the effect sizes were relatively weak (Table S3). All other variables in the biogeographic, climatic and local models had 95% credible intervals that overlapped zero.

### 3.2 Species Analyses

**FIGURE 3:**
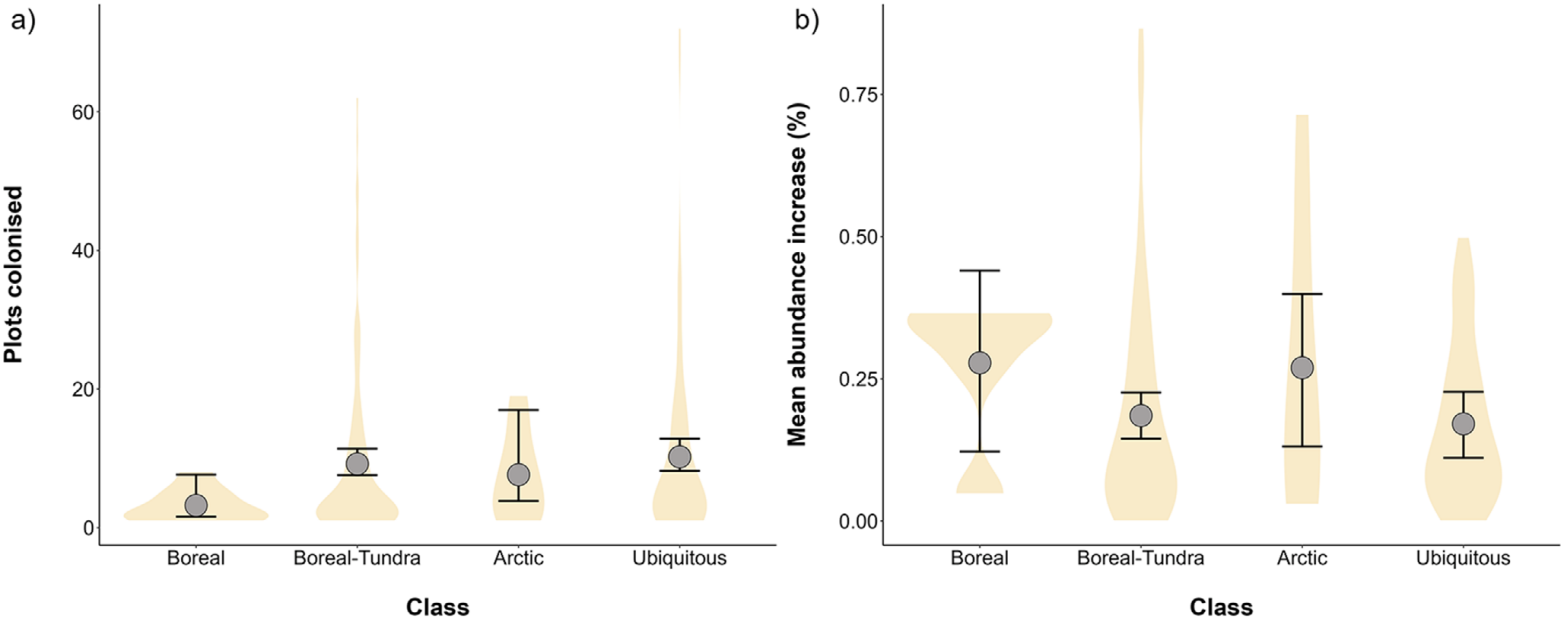
Model estimates at the species level, with (a) total number of times colonising plots (model sample size = 220) and (b) mean annual abundance increases across all plots (model sample size = 129), as a function of class. Violin plots indicate the distribution of the raw values. Points indicate the mean model estimate for each class and error bars the 95% credible intervals. Sample sizes for categories in (a) are: Boreal = 9, Boreal‐Tundra = 113, Arctic = 9, Ubiquitous = 89 species. Sample sizes for categories in (b) are: Boreal = 5, Boreal‐Tundra = 77, Arctic = 7, Ubiquitous = 40 species.

On average, there were 2.8 and 3.2 times fewer colonisations of Boreal than Boreal‐Tundra and Ubiquitous species, respectively, while Arctic species colonisations were very variable (Figure 3a). However, the magnitude of species abundance increases did not differ across different classes and was highly variable for Boreal and Arctic species (Figure 3b). The top coloniser species were the Ubiquitous shrub *Empetrum nigrum* (72 times), the Ubiquitous forb *Persicaria vivipara* (67 times) and the Boreal‐Tundra graminoid *Carex bigelowii* (62 times). Of all species colonising new plots, 64.4% were present within the subsite at the start of monitoring. The species that increased most in abundance include the Boreal‐Tundra shrub *Salix arctophila* (0.87% cover change per year) and the Boreal‐Tundra forbs *Galium verum* (0.83% cover change per year) and *Boykinia richardsonii* (0.77% cover change per year). Generally, species with greater increases in abundance were those that had colonised more frequently over time (slope = 0.75, CI = −0.02 to 1.52), but there was wide variation across species and rates (Table S4). Additionally, species that were gained more frequently were also lost more often (slope = 0.66, CI = 0.59 to 0.72).

**FIGURE 4:**
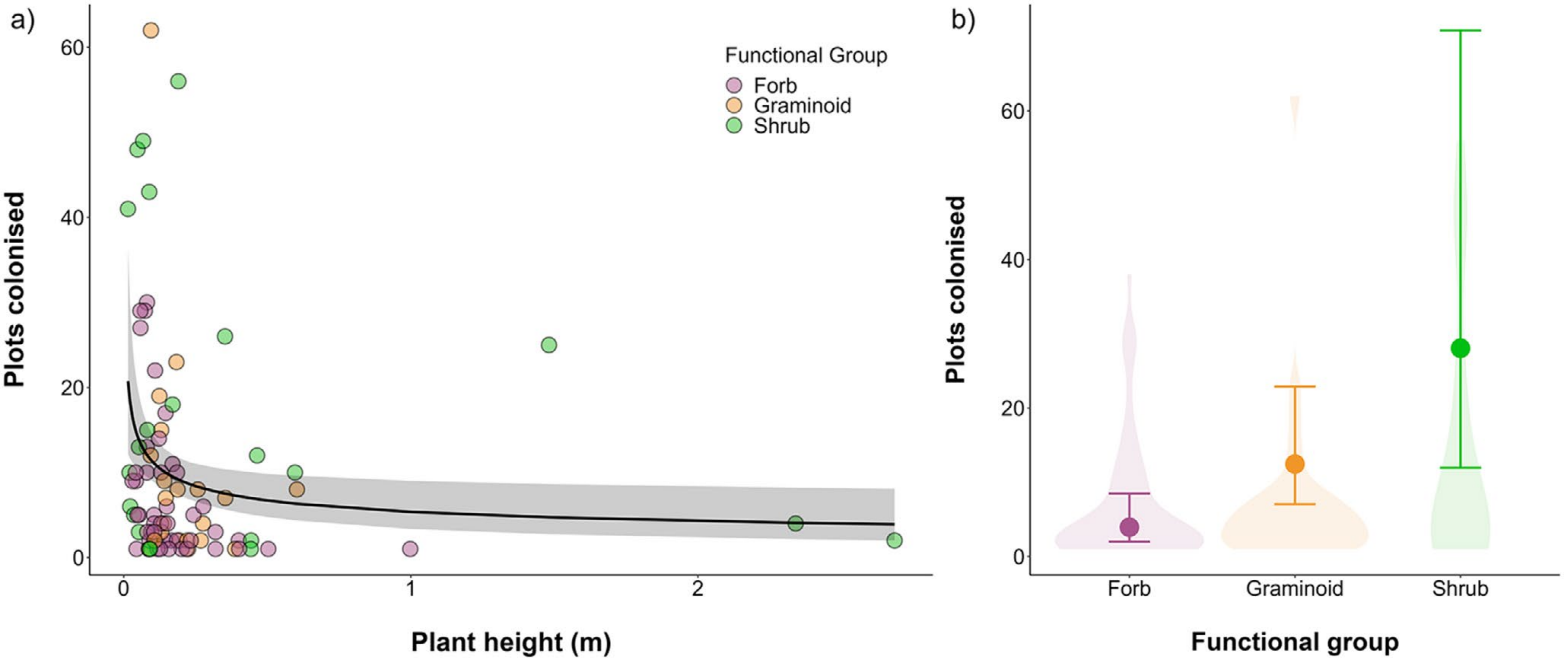
Colonising boreal species were shorter and more likely to be shrubs or graminoids, though shrub species spanned the full range of height values. (a) Boreal species that were shorter colonised plots more often than taller species. Each point represents a species, coloured according to the functional group. The line and ribbon represent the model estimate and 95% credible intervals of the univariate model (to allow for illustration of all the available height values). (b) Boreal shrubs and graminoids colonised more often than forbs. Violin plots indicate the distribution of the raw values. Model outputs are represented as the mean estimate (points) and the 95% credible intervals (error bars). Sample sizes for each category in the model are: forb = 62, graminoid = 32, shrub = 28 species.

Boreal species that colonised and increased in abundance were associated with different traits. Plant height influenced boreal colonisations: shorter species colonised more plots than taller species in the multivariate model (Figure 4a), although they were also likely to be lost from plots more often (slope = −0.72, CI = −1.18 to −0.25). Boreal graminoids and shrubs colonised 3.2 and 7.2 times more than forbs, respectively, in the multivariate model (Figure 4b), while in the univariate model only shrubs colonised 2.1 times more often than forbs (shrub estimate = 0.76, CI = 0.28 to 1.26). Graminoids and shrubs were also lost more frequently from plots (graminoid estimate = 1.97, CI = 1.11 to 2.87, shrub estimate = 2.57, CI = 1.36 to 3.90). There were no other significant traits associated with times colonised in the multivariate model, but in the univariate model species that colonised more often were associated with lower SLA (slope = −0.72, CI = −1.22 to −0.21). Species abundance increases were not associated with any of the five traits tested in either the multivariate or in the univariate models (Table S3). Proportions of boreal coloniser and expanding species per functional group reflected those of the main dataset (i.e., including all species; *z*‐tests, *p* = 0.26 for colonising species and *p* = 0.15 for increasing boreal abundance).

## 4 Discussion

Despite net borealisation being limited and borealisation rates not being different to random expectation, borealisation occurred in around half of the plots, with 52.6% and 42.9% of plots experiencing colonisations by and increases in abundance of boreal species, respectively (Figure 1). These figures align closely with Harsch et al. (2009) and Rees et al. (2020), who reported treeline advance at 52% of studied sites and forest‐tundra ecotone advance at 52.3% of sites, respectively. Boreal expansions were more likely to occur closer to treeline, at warmer and wetter sites and at higher elevations (BCI), and at higher elevation sites and those with lower initial abundance of boreal species (BAI). Within plots with positive rates, boreal increases were greatest at warmer sites (BCI) that had experienced limited precipitation increases (BCI and BAI) and temperature increases (BCI), at sites closer to the treeline, with lower initial abundance of boreal species (BAI), and in Eurasia relative to Greenland‐Iceland (BCI) and Western North America (BAI; Figure 2). Boreal species that contributed most towards borealisation were those that had ranges extending into tundra species pools (i.e., Boreal‐Tundra species; Figure 3). Boreal species that colonised more frequently were shorter and were more likely to be shrubs or graminoids than forbs (Figure 4).

### 4.1 Biogeography, Climate and Local Conditions Affected Borealisation

Our results indicate that borealisation is strongly influenced by biogeoclimatic factors. Boreal colonisations were more likely to occur closer to treeline (Figure 2b,g,i,k), highlighting the role of boreal ecosystems as a current and future source of potentially expanding species into the tundra (Lloyd et al. 2002). Increased boreal abundance and colonisations were more likely to occur at high elevations, suggesting that borealisation is more likely in Oro‐Arctic alpine regions, which have shorter distances to treeline and reduced geographical barriers to dispersal (Chan et al. 2024), rather than in lowland Arctic areas. Regionally, Eurasia experienced greater boreal increases than other regions, in line with modelling studies projecting a more pronounced movement of the taiga‐tundra ecotone in Russia, among other regions (Tang et al. 2023). While the degree of borealisation was highly variable across plant communities (Figure 1, Figure S4), plots that became more ‘borealised’ (both via species colonisations and abundance increases) tended to be situated where climate had changed least (Figure 2e,f,j), although these effect sizes were relatively weak. Generally, Arctic sites with reduced climate change would be closer to the latitudinal treeline, since the Lower Arctic has experienced lower warming rates than the High Arctic (IPCC 2021; this positive relationship between latitude and warming was also present in our dataset: slope = 0.04, 95% CI = 0.01 to 0.07). More colonisations at warmer and wetter sites (Figure 2c,d) suggest that these conditions provide more favourable conditions for Boreal‐Tundra plant establishment, as shown by experimental (Lynn et al. 2021) and observational studies (Dial et al. 2022; García Criado et al. 2020; Roland et al. 2021). Further, plots that experienced greater increases in boreal abundance had lower initial abundance of boreal plants (Figure 2l), indicating greater available niche space for boreal species to expand (Valladares et al. 2015). These findings suggest that borealisation will occur not only close to boreal forests, but also where environmental and local conditions are more conducive to plant establishment.

### 4.2 Boreal Colonisations Were Driven by Boreal‐Tundra Species and by Shrubs and Graminoids

We found that the boreal species contributing most to borealisation were generally of short stature (Figure 4a) and more likely to be shrubs or graminoids than forbs (Figure 4b). This first result ran counter to our expectation that taller plants would be better colonisers, but shorter plants might be better able to cope with colder conditions, including frost and high wind speed (Bliss 1962), and thus relatively small boreal species might be able to succeed in harsher environments (Lynn et al. 2023). Conversely, in line with our hypothesis, shrubs and graminoids colonised more frequently than forbs (Figure 4b), possibly due to their inherent competitive advantages (Bråthen et al. 2021; Liu et al. 2018; Pajunen et al. 2011). Graminoids have deeper root networks and take up nutrients from the soil more readily, thus displacing shallow‐rooted forb and shrub species. However, it should be noted that these traits were also associated with greater species losses, and might indicate that the species driving borealisation are generally more dynamic and contribute more to community turnover rates. Overall, boreal colonisations seem to be driven by relatively more competitive species groups such as shrubs and graminoids, but also by shorter species that might be better adapted to Arctic environmental conditions.

While abundance change was similar across species classes (Figure 3b), Boreal species colonised plots 2.8 and 3.2 fewer times than Boreal–Tundra and Ubiquitous species (Figure 3a). This could indicate that species that are already present in local Arctic species pools (e.g., Boreal‐Tundra, Ubiquitous) are better able to establish in new areas (e.g., through propagule pressure). Widespread species might be generalists and/or have broader bioclimatic niches, and could therefore increase in abundance and ultimately expand into new areas more efficiently than purely Boreal species (Timoney 2023), mirroring European trends with large‐range species driving turnover (Staude et al. 2022). Species with populations that currently exist in the tundra likely have characteristics that allow them to survive in harsher environmental conditions, while Boreal species might not survive these environmental filters towards successful dispersal and establishment (Callaghan et al. 2004a). For instance, boreal understory shade‐adapted species could struggle with greater light availability in open tundra habitats and their greater temperature fluctuations (De Pauw et al. 2022; Sanczuk et al. 2023), thus leading to reduced expansions of Boreal specialists in the tundra. Therefore, purely Boreal species might be limited in their abilities to colonise new tundra ecosystems.

The influx of boreal species could be intertwined with ‘hidden diversity’ as one of the main pathways of future community composition change. Hidden diversity refers to species present in local species pools that have not yet reached long‐term monitoring plots (Pärtel 2014). With 64.4% of plot colonising species being present at the subsite level at the start of monitoring, many local colonisations were from species with a widespread distribution, which have the greatest potential to increase their abundance and distribution under climate change, rather than species migrating from the boreal forest (Callaghan et al. 2004a; Timoney 2023). Overall, our results indicate that borealisation will likely be driven by widespread species with ranges extending into the Arctic that are present in local species pools, and not by Boreal species alone.

### 4.3 Considerations

Borealisation is a process with multiple interacting variables. Here, we have removed correlated variables by quantifying the relationships between potential drivers (Figures S2 and S3). There are certain considerations associated with field methods such as point‐framing, where some present species could be missed during surveys and result in artificial colonisations and/or losses. While it remains important to design field studies to account for species detectability (Kéry 2004), the point‐framing method has been generally considered to result in an accurate representation of plant communities (May and Hollister 2012). Acknowledging that ‘top coloniser’ species (Table S4) were also lost more frequently from plots because they are locally abundant within the landscape or they have high turnover rates (Staude et al. 2022), we focus here on colonisations and abundance increases to better understand the borealisation process. Relatedly, our null models showed that the observed values of BCI and BAI were not different from those that would be expected by chance alone, thus reflecting actual plant community dynamics. Here, we focus on borealisation as one such process of community change and aim at capturing its occurrence and associated drivers.

While in this study we focus on the process of borealisation, negative borealisation rates were observed at some sites (Figure S4), indicating a loss or reduced abundance of boreal species. Negative, slow, or lagged rates of borealisation may be due to stochastic processes and other factors that limit plant community change (Lenoir et al. 2020). For example, (1) migration lags of plants into the Arctic at millennial scales have been reported following deglaciation periods (Alsos et al. 2022). (2) Herbivory can dampen species turnover (Speed et al. 2012), although herbivory was not a significant predictor of borealisation in our analyses. This could be due to the coarse scale subsite‐level herbivory information of the ITEX experimental design (Barrio et al. 2022). (3) Some species, including conifers (typically boreal species), have slow establishment rates, particularly at their northern range edges (Kroiss and HilleRisLambers 2015). (4) Plant recruitment and survival are highly sensitive to interannual climatic variation (Harsch et al. 2009), while climate‐derived effects on reproduction can limit boreal treeline expansion (Brown et al. 2019). (5) Since our study sites are often far from the treeline and not random in their distribution, this could explain the low number of Boreal species within our dataset. Thus, more standardised studies at the boreal–tundra ecotone boundary are needed to refine estimates of boreal species encroachment rates. Future studies could characterise borealisation at the landscape scale rather than plot scale, which might reveal somewhat different drivers.

## 5 Conclusions

Together, our findings suggest that future changes to tundra ecosystems might not involve rapid biome shifts where boreal species replace tundra species. Rather, we might expect an overall increase in the presence and abundance of species with broader geographic ranges that were already present within the Arctic, leading to a slower boreal–tundra ecotone displacement into the tundra biome. Looking forward, borealisation could occur not only at sites near the treeline, but also where Boreal–Tundra species are present in Arctic species pools. This borealisation of tundra plant communities will have implications for wildlife habitats, trophic interactions, ecosystem functions and people living in the Arctic. If tundra plant communities become more boreal, this could expand habitat for boreal herbivores such as Middendorff's vole (*Alexandromys middendorffii*), beaver ( *Castor canadensis* ) and moose (Tape et al. 2018; Sokolova et al. 2024; Zhou et al. 2020). Simultaneously, borealisation could reduce habitat quality for tundra species such as barren‐ground caribou ( *Rangifer tarandus groenlandicus* ; Fullman et al. 2017), which feed mostly on lichen that could be outcompeted by shrub growth (Cornelissen et al. 2001). However, climate itself could be a stronger driver of herbivore community change (Speed et al. 2021). Increased presence of boreal plant species within the tundra could also alter nutrient cycling and reduce soil carbon storage (Gustafson et al. 2021; Parker et al. 2021; Speed et al. 2015), while also decreasing albedo, thus leading to further permafrost thaw and creating positive climate feedbacks via increased warming (Heijmans et al. 2022). Ultimately, borealisation could also pose socio‐economic impacts for people, for instance compromising food security through shifts in edible plants and altered migration routes for reindeer/caribou (Hupp et al. 2015; Rees et al. 2008). Overall, future research is needed to unveil the multi‐faceted consequences of tundra borealisation.

## Author Contributions

M.G.C. conceived the study together with I.C.B. and J.D.M.S., with conceptual contributions from I.H.M.S. and A.D.B. M.G.C., A.D.B. and S.C.E. compiled and cleaned the ITEX+ plant composition data. M.G.C., I.C.B. and S.C.E. produced the visualisations. M.G.C. and I.C.B. undertook the analyses and M.G.C. wrote the manuscript, with contributions from all authors. I.H.M.S., R.A., J.M.A., K.R.B.‐M., R.G.B., M.P.B., D.B., E.J.C., J.H.C.C., S.C.E., W.A.G., R.G., G.H.R.H., L.H., R.D.H., A.K.J., I.S.J., E.K., O.K., S.I.L., P.M., J.L.M., A.M., S.N., S.L.O., E.P., R.R., N.M.S., S.S., A.T., J.P.T., A.T., V.V. and T.V. collected the ITEX+ plant composition data.

## Peer Review

The peer review history for this article is available at https://www.webofscience.com/api/gateway/wos/peer‐review/10.1111/ele.70209.

## Supporting information


**Appendix S1:** ele70209‐sup‐0001‐supinfo.pdf.

## Data Availability

Data and code are available in Zenodo at https://doi.org/10.5281/zenodo.14960354.

## References

[ele70209-bib-0001] Aarnes, I. , A. E. Bjune , H. H. Birks , N. L. Balascio , J. Bakke , and M. Blaauw . 2012. “Vegetation Responses to Rapid Climatic Changes During the Last Deglaciation 13,500–8,000 Years Ago on Southwest Andøya, Arctic Norway.” Vegetation History and Archaeobotany 21: 17–35.

[ele70209-bib-0002] Abbott, R. J. , L. C. Smith , R. I. Milne , R. M. M. Crawford , K. Wolff , and J. Balfour . 2000. “Molecular Analysis of Plant Migration and Refugia in the Arctic.” Science 289: 1343–1346. 10958779 10.1126/science.289.5483.1343

[ele70209-bib-0003] Alsos, I. G. , D. P. Rijal , D. Ehrich , et al. 2022. “Postglacial Species Arrival and Diversity Buildup of Northern Ecosystems Took Millennia.” Science Advances 8: eabo7434. 36170372 10.1126/sciadv.abo7434 PMC9519041

[ele70209-bib-0004] Aubin, I. , A. D. Munson , F. Cardou , et al. 2016. “Traits to Stay, Traits to Move: A Review of Functional Traits to Assess Sensitivity and Adaptive Capacity of Temperate and Boreal Trees to Climate Change.” Environmental Reviews 24: 164–186.

[ele70209-bib-0005] Barrio, I. C. , D. Ehrich , E. M. Soininen , et al. 2022. “Developing Common Protocols to Measure Tundra Herbivory Across Spatial Scales.” Arctic Science 8: 638–679.

[ele70209-bib-0006] Behrend, A. M. , Á. L. Aradóttir , K. Svavarsdóttir , T. E. Thórhallsdóttir , and A. Pommerening . 2024. “Natural Colonization as a Means to Upscale Restoration of Subarctic Woodlands in Iceland.” Restoration Ecology 33: e14332.

[ele70209-bib-0007] Birks, H. H. 2008. “The Late‐Quaternary History of Arctic and Alpine Plants.” Plant Ecology and Diversity 1: 135–146.

[ele70209-bib-0008] Bjorkman, A. D. , I. H. Myers‐Smith , S. C. Elmendorf , et al. 2018a. “Plant Functional Trait Change Across a Warming Tundra Biome.” Nature 562: 57–62. 30258229 10.1038/s41586-018-0563-7

[ele70209-bib-0009] Bjorkman, A. D. , I. H. Myers‐Smith , S. C. Elmendorf , et al. 2018b. “Tundra Trait Team: A Database of Plant Traits Spanning the Tundra Biome.” Global Ecology and Biogeography 27: 1402–1411.

[ele70209-bib-0010] Bliss, L. C. 1962. “Adaptations of Arctic and Alpine Plants to Environmental Conditions.” Arctic 15: 117–144.

[ele70209-bib-0011] Bråthen, K. A. , F. I. Pugnaire , and R. D. Bardgett . 2021. “The Paradox of Forbs in Grasslands and the Legacy of the Mammoth Steppe.” Frontiers in Ecology and the Environment 19: 584–592.

[ele70209-bib-0012] Brown, C. D. , G. Dufour‐Tremblay , R. G. Jameson , et al. 2019. “Reproduction as a Bottleneck to Treeline Advance Across the Circumarctic Forest Tundra Ecotone.” Ecography 42: 137–147.

[ele70209-bib-0013] Bürkner, P.‐C. 2017. “brms: An R Package for Bayesian Multilevel Models Using Stan.” Journal of Statistical Software 80: 1–28.

[ele70209-bib-0014] Callaghan, T. V. , L. O. Björn , Y. Chernov , et al. 2004a. “Biodiversity, Distributions and Adaptations of Arctic Species in the Context of Environmental Change.” Ambio 33: 404–417. 15573569 10.1579/0044-7447-33.7.404

[ele70209-bib-0015] Callaghan, T. V. , L. O. Björn , Y. Chernov , et al. 2004b. “Synthesis of Effects in Four Arctic Subregions.” Ambio 33: 469–473. 15573574 10.1579/0044-7447-33.7.469

[ele70209-bib-0016] Callaghan, T. V. , B. R. Werkman , and R. M. M. Crawford . 2002. “The Tundra‐Taiga Interface and Its Dynamics: Concepts and Applications.” Ambio 12: 6–14. 12374061

[ele70209-bib-0017] Chan, W. P. , J. Lenoir , G. S. Mai , H. C. Kuo , I. C. Chen , and S. F. Shen . 2024. “Climate Velocities and Species Tracking in Global Mountain Regions.” Nature 629, no. 8010: 114–120. 38538797 10.1038/s41586-024-07264-9 PMC11062926

[ele70209-bib-0018] Cornelissen, J. H. C. , T. V. Callaghan , J. M. Alatalo , et al. 2001. “Global Change and Arctic Ecosystems: Is Lichen Decline a Function of Increases in Vascular Plant Biomass?” Journal of Ecology 89, no. 6: 984–994.

[ele70209-bib-0019] Daniëls, F. J. A. , L. J. Gillespie , and M. Poulin . 2013. “Chapter 9. Plants.” In Arctic Biodiversity Assessment. Status and Trends in Arctic Biodiversity, 311–353. Conservation of Arctic Flora and Fauna.

[ele70209-bib-0020] De Pauw, K. , P. Sanczuk , C. Meeussen , et al. 2022. “Forest Understorey Communities Respond Strongly to Light in Interaction With Forest Structure, but Not to Microclimate Warming.” New Phytologist 233, no. 1: 219–235. 34664731 10.1111/nph.17803

[ele70209-bib-0021] Dial, R. J. , C. T. Maher , R. E. Hewitt , and P. F. Sullivan . 2022. “Sufficient Conditions for Rapid Range Expansion of a Boreal Conifer.” Nature 608: 546–551. 35948635 10.1038/s41586-022-05093-2 PMC9385489

[ele70209-bib-0022] Dunn, P. K. , and G. K. Smyth . 2018. “Chapter 9: Models for Proportions: Binomial GLMs.” In Generalized Linear Models With Examples in R, edited by P. K. Dunn and G. K. Smyth , 333–369. Springer.

[ele70209-bib-0023] Elmendorf, S. C. , G. H. R. Henry , R. D. Hollister , et al. 2012. “Plot‐Scale Evidence of Tundra Vegetation Change and Links to Recent Summer Warming.” Nature Climate Change 2: 453–457.

[ele70209-bib-0024] Elmendorf, S. C. , G. H. R. Henry , R. D. Hollister , et al. 2015. “Experiment, Monitoring, and Gradient Methods Used to Infer Climate Change Effects on Plant Communities Yield Consistent Patterns.” Proceedings of the National Academy of Sciences of the United States of America 112: 448–452. 25548195 10.1073/pnas.1410088112 PMC4299205

[ele70209-bib-0025] Elmhagen, B. , D. Berteaux , R. M. Burgess , et al. 2017. “Homage to Hersteinsson and Macdonald: Climate Warming and Resource Subsidies Cause Red Fox Range Expansion and Arctic Fox Decline.” Polar Research 36: 3.

[ele70209-bib-0026] Elven, R. 2007. Checklist of the Panarctic Flora (PAF) Vascular Plants. National Centre of Biosystematics, Natural History Museum, University of Oslo.

[ele70209-bib-0027] Emblemsvåg, M. , L. Pecuchet , L. G. Velle , A. Nogueira , and R. Primicerio . 2022. “Recent Warming Causes Functional Borealization and Diversity Loss in Deep Fish Communities East of Greenland.” Diversity and Distributions 28: 2071–2083.

[ele70209-bib-0028] Eskelinen, A. , E. Kaarlejärvi , and J. Olofsson . 2017. “Herbivory and Nutrient Limitation Protect Warming Tundra From Lowland Species' Invasion and Diversity Loss.” Global Change Biology 23: 245–255. 27343482 10.1111/gcb.13397

[ele70209-bib-0029] Fauchald, P. , T. Park , H. Tommervik , R. Myneni , and V. H. Hausner . 2017. “Arctic Greening From Warming Promotes Declines in Caribou Populations.” Science Advances 3, no. 4: e1601365. 28508037 10.1126/sciadv.1601365 PMC5406139

[ele70209-bib-0030] Frost, G. V. , and H. E. Epstein . 2014. “Tall Shrub and Tree Expansion in Siberian Tundra Ecotones Since the 1960s.” Global Change Biology 20: 1264–1277. 24115456 10.1111/gcb.12406

[ele70209-bib-0031] Fullman, T. J. , K. Joly , and A. Ackerman . 2017. “Effects of Environmental Features and Sport Hunting on Caribou Migration in Northwestern Alaska.” Movement Ecology 5: 4. 28270913 10.1186/s40462-017-0095-z PMC5331706

[ele70209-bib-0032] García Criado, M. , I. H. Myers‐Smith , A. D. Bjorkman , et al. 2025. “Plant Diversity Dynamics Over Space and Time in a Warming Arctic.” Nature 642: 653–661. 40307554 10.1038/s41586-025-08946-8 PMC12176628

[ele70209-bib-0033] García Criado, M. , I. H. Myers‐Smith , A. D. Bjorkman , C. E. R. Lehmann , and N. Stevens . 2020. “Woody Plant Encroachment Intensifies Under Climate Change Across Tundra and Savanna Biomes.” Global Ecology and Biogeography 29: 925–943.

[ele70209-bib-0034] García Criado, M. , I. H. Myers‐Smith , A. D. Bjorkman , et al. 2023. “Plant Traits Poorly Predict Winner and Loser Shrub Species in a Warming Tundra Biome.” Nature Communications 14: 3837. 10.1038/s41467-023-39573-4 PMC10307830 37380662

[ele70209-bib-0035] GBIF . 2024. “GBIF.org.”

[ele70209-bib-0036] Graae, B. J. , V. Vandvik , W. S. Armbruster , et al. 2018. “Stay or Go—How Topographic Complexity Influences Alpine Plant Population and Community Responses to Climate Change.” Perspectives in Plant Ecology, Evolution and Systematics 30: 41–50.

[ele70209-bib-0037] Gustafson, A. , P. A. Miller , R. G. Björk , S. Olin , and B. Smith . 2021. “Nitrogen Restricts Future Sub‐Arctic Treeline Advance in an Individual‐Based Dynamic Vegetation Model.” Biogeosciences 18: 6329–6347.

[ele70209-bib-0038] Harris, J. A. , R. D. Hollister , T. F. Botting , et al. 2022. “Understanding the Climate Impacts on Decadal Vegetation Change in Northern Alaska.” Arctic Science 8: 878–898.

[ele70209-bib-0039] Harsch, M. A. , P. E. Hulme , M. S. McGlone , and R. P. Duncan . 2009. “Are Treelines Advancing? A Global Meta‐Analysis of Treeline Response to Climate Warming.” Ecology Letters 12: 1040–1049. 19682007 10.1111/j.1461-0248.2009.01355.x

[ele70209-bib-0040] Heijmans, M. M. , R. Í. Magnússon , M. J. Lara , et al. 2022. “Tundra Vegetation Change and Impacts on Permafrost.” Nature Reviews Earth and Environment 3, no. 1: 68–84.

[ele70209-bib-0041] Henry, G. H. R. , and U. Molau . 1997. “Tundra Plants and Climate Change: The International Tundra Experiment (ITEX).” Global Change Biology 3: 1–9.

[ele70209-bib-0042] Hupp, J. , M. Brubaker , K. Wilkinson , and J. Williamson . 2015. “How Are Your Berries? Perspectives of Alaska's Environmental Managers on Trends in Wild Berry Abundance.” International Journal of Circumpolar Health 74: 28704. 26380964 10.3402/ijch.v74.28704 PMC4574151

[ele70209-bib-0043] IPCC . 2021. Climate Change 2021: The Physical Science Basis. Contribution of Working Group I to the Sixth Assessment Report of the Intergovernmental Panel on Climate Change. Cambridge University Press.

[ele70209-bib-0044] Kaarlejärvi, E. , A. Eskelinen , and J. Olofsson . 2017. “Herbivores Rescue Diversity in Warming Tundra by Modulating Trait‐Dependent Species Losses and Gains.” Nature Communications 8: 419. 10.1038/s41467-017-00554-z PMC5583392 28871154

[ele70209-bib-0045] Karger, D. N. , O. Conrad , J. Böhner , et al. 2017. “Climatologies at High Resolution for the Earth's Land Surface Areas.” Scientific Data 4: 170122. 28872642 10.1038/sdata.2017.122 PMC5584396

[ele70209-bib-0046] Kattge, J. , G. Bönisch , S. Díaz , et al. 2020. “TRY Plant Trait Database—Enhanced Coverage and Open Access.” Global Change Biology 26: 119–188. 31891233 10.1111/gcb.14904

[ele70209-bib-0047] Kéry, M. 2004. “Extinction Rate. Estimates for Plant Populations in Revisitation Studies: Importance of Detectability.” Conservation Biology 18, no. 2: 570–574.

[ele70209-bib-0048] Khitun, O. V. , T. M. Koroleva , S. V. Chinenko , et al. 2016. “Applications of Local Floras for Floristic Subdivision and Monitoring Vascular Plant Diversity in the Russian Arctic.” Arctic Science 2: 103–126.

[ele70209-bib-0049] Kindt, R. 2020. “WorldFlora: An R Package for Exact and Fuzzy Matching of Plant Names Against the World Flora Online Taxonomic Backbone Data.” Applications in Plant Sciences 8: e11388. 33014632 10.1002/aps3.11388 PMC7526431

[ele70209-bib-0050] Kroiss, S. J. , and J. HilleRisLambers . 2015. “Recruitment Limitation of Long‐Lived Conifers: Implications for Climate Change Responses.” Ecology 96: 1286–1297. 26236842 10.1890/14-0595.1

[ele70209-bib-0051] Le Pogam, A. , R. S. O'Connor , O. P. Love , M. Petit , L. Régimbald , and F. Vézina . 2021. “Coping With the Worst of Both Worlds: Phenotypic Adjustments for Cold Acclimatization Benefit Northward Migration and Arrival in the Cold in an Arctic‐Breeding Songbird.” Functional Ecology 35: 1240–1254.

[ele70209-bib-0052] Lenoir, J. , R. Bertrand , L. Comte , et al. 2020. “Species Better Track Climate Warming in the Oceans Than on Land.” Nature Ecology & Evolution 4: 1044–1059. 32451428 10.1038/s41559-020-1198-2

[ele70209-bib-0053] Liu, H. , Z. Mi , L. Lin , et al. 2018. “Shifting Plant Species Composition in Response to Climate Change Stabilizes Grassland Primary Production.” Proceedings of the National Academy of Sciences of the United States of America 115: 4051–4056. 29666319 10.1073/pnas.1700299114 PMC5910805

[ele70209-bib-0054] Lloyd, A. H. , T. S. Rupp , C. L. Fastie , and A. M. Starfield . 2002. “Patterns and Dynamics of Treeline Advance on the Seward Peninsula, Alaska.” Journal of Geophysical Research: Atmospheres 107: ALT 2‐1–ALT 2‐15.

[ele70209-bib-0055] Lynn, J. S. , R. Gya , K. Klanderud , R. J. Telford , D. E. Goldberg , and V. Vandvik . 2023. “Traits Help Explain Species' Performance Away From Their Climate Niche Centre.” Diversity and Distributions 29: 962–978.

[ele70209-bib-0056] Lynn, J. S. , K. Klanderud , R. J. Telford , D. E. Goldberg , and V. Vandvik . 2021. “Macroecological Context Predicts Species' Responses to Climate Warming.” Global Change Biology 27: 2088–2101. 33511713 10.1111/gcb.15532

[ele70209-bib-0057] Macias‐Fauria, M. , B. C. Forbes , P. Zetterberg , and T. Kumpula . 2012. “Eurasian Arctic Greening Reveals Teleconnections and the Potential for Novel Ecosystems.” Environmental Reviews 2: 613–618.

[ele70209-bib-0058] Mallory, C. D. , and M. S. Boyce . 2018. “Observed and Predicted Effects of Climate Change on Arctic Caribou and Reindeer.” Environmental Reviews 26: 13–25.

[ele70209-bib-0059] May, J. L. , and R. D. Hollister . 2012. “Validation of a Simplified Point Frame Method to Detect Change in Tundra Vegetation.” Polar Biology 35: 1815–1823.

[ele70209-bib-0060] Meltofte, H. 2013. Arctic Biodiversity Assessment. Status and Trends in Arctic Biodiversity. Conservation of Arctic Flora and Fauna.

[ele70209-bib-0061] Molau, U. , and P. Mølgaard , eds. 1996. ITEX Manual. ITEX Secretariat.

[ele70209-bib-0062] Myers‐Smith, I. H. , B. C. Forbes , M. Wilmking , et al. 2011. “Shrub Expansion in Tundra Ecosystems: Dynamics, Impacts and Research Priorities.” Environmental Research Letters 6: 045509.

[ele70209-bib-0063] Obu, J. , S. Westermann , A. Bartsch , et al. 2019. “Northern Hemisphere Permafrost Map Based on TTOP Modelling for 2000–2016 at 1 km^2^ Scale.” Earth‐Science Reviews 193: 299–316.

[ele70209-bib-0064] Olson, D. M. , E. Dinerstein , E. D. Wikramanayake , et al. 2001. “Terrestrial Ecoregions of the World: A New Map of Life on Earth: A New Global Map of Terrestrial Ecoregions Provides an Innovative Tool for Conserving Biodiversity.” Bioscience 51: 933–938.

[ele70209-bib-0065] Pajunen, A. M. , J. Oksanen , and R. Virtanen . 2011. “Impact of Shrub Canopies on Understorey Vegetation in Western Eurasian Tundra.” Journal of Vegetation Science 22: 837–846.

[ele70209-bib-0066] Parker, T. C. , A. M. Thurston , K. Raundrup , J.‐A. Subke , P. A. Wookey , and I. P. Hartley . 2021. “Shrub Expansion in the Arctic May Induce Large‐Scale Carbon Losses due to Changes in Plant‐Soil Interactions.” Plant and Soil 463: 643–651.

[ele70209-bib-0067] Pärtel, M. 2014. “Community Ecology of Absent Species: Hidden and Dark Diversity.” Journal of Vegetation Science 25: 1154–1159.

[ele70209-bib-0068] Pecuchet, L. , M.‐A. Blanchet , A. Frainer , et al. 2020. “Novel Feeding Interactions Amplify the Impact of Species Redistribution on an Arctic Food Web.” Global Change Biology 26: 4894–4906. 32479687 10.1111/gcb.15196

[ele70209-bib-0069] R Core Team . 2022. R: A Language and Environment for Statistical Computing. R Foundation for Statistical Computing.

[ele70209-bib-0070] Ray, N. , and J. Adams . 2001. “A GIS‐Based Vegetation Map of the World at the Last Glacial Maximum (25,000–15,000 BP).” Internet Archaeology 11: 1–44.

[ele70209-bib-0071] Raynolds, M. K. , D. A. Walker , A. Balser , et al. 2019. “A Raster Version of the Circumpolar Arctic Vegetation Map (CAVM).” Remote Sensing of Environment 232: 111297.

[ele70209-bib-0072] Rees, W. G. , A. Hofgaard , S. Boudreau , et al. 2020. “Is Subarctic Forest Advance Able to Keep Pace With Climate Change?” Global Change Biology 26: 3965–3977. 32281711 10.1111/gcb.15113

[ele70209-bib-0073] Rees, W. G. , F. M. Stammler , F. S. Danks , and P. Vitebsky . 2008. “Vulnerability of European Reindeer Husbandry to Global Change.” Climatic Change 87: 199–217.

[ele70209-bib-0074] Roland, C. , J. H. Schmidt , S. E. Stehn , C. J. Hampton‐Miller , and E. F. Nicklen . 2021. “Borealization and Its Discontents: Drivers of Regional Variation in Plant Diversity Across Scales in Interior Alaska.” Ecosphere 12: e03485.

[ele70209-bib-0075] Ropars, P. , and S. Boudreau . 2012. “Shrub Expansion at the Forest–Tundra Ecotone: Spatial Heterogeneity Linked to Local Topography.” Environmental Research Letters 7: 015501.

[ele70209-bib-0076] Rupp, T. S. , F. S. Chapin , and A. M. Starfield . 2001. “Modeling the Influence of Topographic Barriers on Treeline Advance at the Forest‐Tundra Ecotone in Northwestern Alaska.” Climatic Change 48: 399–416.

[ele70209-bib-0077] Sanczuk, P. , K. De Pauw , E. De Lombaerde , et al. 2023. “Microclimate and Forest Density Drive Plant Population Dynamics Under Climate Change.” Nature Climate Change 13, no. 8: 840–847.

[ele70209-bib-0078] Sokolova, N. A. , I. A. Fufachev , D. Ehrich , V. G. Shtro , V. A. Sokolov , and A. A. Sokolov . 2024. “Expansion of Voles and Retraction of Lemmings Over 60 Years Along a Latitudinal Gradient on Yamal Peninsula.” Global Change Biology 30, no. 2: e17161.

[ele70209-bib-0079] Speed, J. D. M. , G. Austrheim , A. J. Hester , and A. Mysterud . 2012. “Elevational Advance of Alpine Plant Communities Is Buffered by Herbivory.” Journal of Vegetation Science 23: 617–625.

[ele70209-bib-0080] Speed, J. D. M. , J. A. Chimal‐Ballesteros , M. D. Martin , I. C. Barrio , K. E. M. Vuorinen , and E. M. Soininen . 2021. “Will Borealization of Arctic Tundra Herbivore Communities Be Driven by Climate Warming or Vegetation Change?” Global Change Biology 27: 6568–6577. 34592044 10.1111/gcb.15910

[ele70209-bib-0081] Speed, J. D. M. , V. Martinsen , A. J. Hester , et al. 2015. “Continuous and Discontinuous Variation in Ecosystem Carbon Stocks With Elevation Across a Treeline Ecotone.” Biogeosciences 12: 1615–1627.

[ele70209-bib-0082] Sporbert, M. , E. Welk , G. Seidler , et al. 2021. “Different Sets of Traits Explain Abundance and Distribution Patterns of European Plants at Different Spatial Scales.” Journal of Vegetation Science 32: e13016.

[ele70209-bib-0083] Staude, I. R. , H. M. Pereira , G. N. Daskalova , et al. 2022. “Directional Turnover Towards Larger‐Ranged Plants Over Time and Across Habitats.” Ecology Letters 25: 466–482. 34866301 10.1111/ele.13937

[ele70209-bib-0084] Steinbauer, M. J. , J.‐A. Grytnes , G. Jurasinski , et al. 2018. “Accelerated Increase in Plant Species Richness on Mountain Summits Is Linked to Warming.” Nature 556: 231–234. 29618821 10.1038/s41586-018-0005-6

[ele70209-bib-0085] Tang, J. , P. Zhou , P. A. Miller , et al. 2023. “High‐Latitude Vegetation Changes Will Determine Future Plant Volatile Impacts on Atmospheric Organic Aerosols.” npj Climate and Atmospheric Science 6: 1–13.

[ele70209-bib-0086] Tape, K. D. , D. D. Gustine , R. W. Ruess , L. G. Adams , and J. A. Clark . 2016. “Range Expansion of Moose in Arctic Alaska Linked to Warming and Increased Shrub Habitat.” PLoS One 11: e0152636. 27074023 10.1371/journal.pone.0152636 PMC4830447

[ele70209-bib-0087] Tape, K. D. , B. M. Jones , C. D. Arp , I. Nitze , and G. Grosse . 2018. “Tundra Be Dammed: Beaver Colonization of the Arctic.” Global Change Biology 24: 4478–4488. 29845698 10.1111/gcb.14332

[ele70209-bib-0088] Thomas, H. J. D. , A. D. Bjorkman , I. H. Myers‐Smith , et al. 2020. “Global Plant Trait Relationships Extend to the Climatic Extremes of the Tundra Biome.” Nature Communications 11: 1–12. 10.1038/s41467-020-15014-4 PMC7067758 32165619

[ele70209-bib-0089] Timoney, K. P. 2023. “No Evidence of a Northward Biome Shift of Treeline in the Mackay Lake Region, North‐Central Canada.” Écoscience 30: 113–129.

[ele70209-bib-0090] Valdez, J. W. , C. T. Callaghan , J. Junker , A. Purvis , S. L. L. Hill , and H. M. Pereira . 2023. “The Undetectability of Global Biodiversity Trends Using Local Species Richness.” Ecography 2023: e06604.

[ele70209-bib-0091] Valladares, F. , C. C. Bastias , O. Godoy , E. Granda , and A. Escudero . 2015. “Species Coexistence in a Changing World.” Frontiers in Plant Science 6: 866. 26528323 10.3389/fpls.2015.00866 PMC4604266

[ele70209-bib-0092] Verdonen, M. , I. C. Barrio , L. Barbero‐Palacios , et al. 2025. “Borealization of Tundra Ecosystems With Climate and Land‐Use Change.” *EcoEvoRxiv*. 10.32942/X2JS9X.

[ele70209-bib-0093] Villén‐Peréz, S. , J. Heikkinen , M. Salemaa , and R. Mäkipää . 2020. “Global Warming Will Affect the Maximum Potential Abundance of Boreal Plant Species.” Ecography 43: 801–811.

[ele70209-bib-0094] Vincent, H. , C. N. Bornand , A. Kempel , and M. Fischer . 2020. “Rare Species Perform Worse Than Widespread Species Under Changed Climate.” Biological Conservation 246: 108586.

[ele70209-bib-0095] Vuorinen, K. E. M. , L. Oksanen , T. Oksanen , A. Pyykonen , J. Olofsson , and R. Virtanen . 2017. “Open Tundra Persist, but Arctic Features Decline‐Vegetation Changes in the Warming Fennoscandian Tundra.” Global Change Biology 23: 3794–3807. 28488280 10.1111/gcb.13710

[ele70209-bib-0096] Walker, D. A. , M. K. Raynolds , F. J. A. Daniëls , et al. 2005. “The Circumpolar Arctic Vegetation Map.” Journal of Vegetation Science 16: 267–282.

[ele70209-bib-0097] Wei, T. , and V. Simko . 2021. R Package ‘corrplot’: Visualization of a Correlation Matrix (Version 0.92). https://github.com/taiyun/corrplot.

[ele70209-bib-0098] Westoby, M. 1998. “A Leaf‐Height‐Seed (LHS) Plant Ecology Strategy Scheme.” Plant and Soil 199: 213–227.

[ele70209-bib-0099] WFO . 2024. “World Flora Online.” https://www.worldfloraonline.org/.

[ele70209-bib-0100] Zhang, W. , P. A. Miller , B. Smith , R. Wania , T. Koenigk , and R. Döscher . 2013. “Tundra Shrubification and Tree‐Line Advance Amplify Arctic Climate Warming: Results From an Individual‐Based Dynamic Vegetation Model.” Environmental Research Letters 8: 034023.

[ele70209-bib-0101] Zhou, J. , K. D. Tape , L. Prugh , G. Kofinas , G. Carroll , and K. Kielland . 2020. “Enhanced Shrub Growth in the Arctic Increases Habitat Connectivity for Browsing Herbivores.” Global Change Biology 26: 3809–3820. 32243648 10.1111/gcb.15104

