# Supplementary material for: Borealisation of Plant Communities in the Arctic Is Driven by Boreal‐Tundra Species
Source: Ecol Lett. 2025 Sep 21;28(9):e70209. doi: 10.1111/ele.70209 (PMC12451259; doi:10.1111/ele.70209)
Supplement: Supplementary file 1 — Appendix S1: ele70209‐sup‐0001‐supinfo.pdf. [file ELE-28-0-s001.pdf]

Borealisation of Plant Communities in the Arctic Is Driven by Boreal-Tundra Species

Supplementary Information

Mariana García Criado, Isabel C. Barrio, James D. M. Speed, Anne D. Bjorkman, Sarah C. Elmendorf, Isla H. Myers-Smith, Rien Aerts, Juha M. Alatalo, Katlyn R. Betway-May, Robert G. Björk, Mats P. Björkman, Daan Blok, Elisabeth J. Cooper, J. Hans C. Cornelissen, William A. Gould, Ragnhild Gya, Greg H.R. Henry, Luise Hermanutz, Robert D. Hollister, Annika K. Jägerbrand, Ingibjörg S. Jónsdóttir, Elina Kaarlejärvi, Olga Khitun, Simone I. Lang, Petr Macek, Jeremy L. May, Anders Michelsen, Signe Normand, Siri L. Olsen, Eric Post, Riikka Rinnan, Niels Martin Schmidt, Sofie Sjogersten, Anne Tolvanen, Joachim P. Töpper, Andrew Trant, Vigdis Vandvik and Tage Vowles

Supplementary Figures.

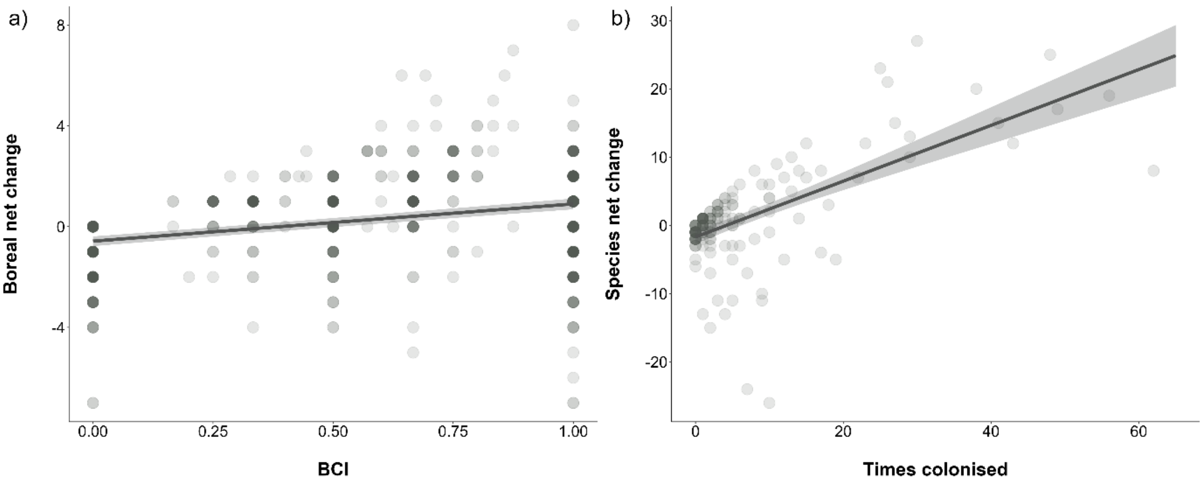

Figure S1. There were positive correlations between BCI and net change values at the community level (**a**) and between times colonised and net change at the species level (**b**). Darker grey colours indicate overlap of multiple points. Solid lines represent the model estimate, with credible intervals that did not overlap zero. Semi-transparent ribbons represent the 95% credible intervals.

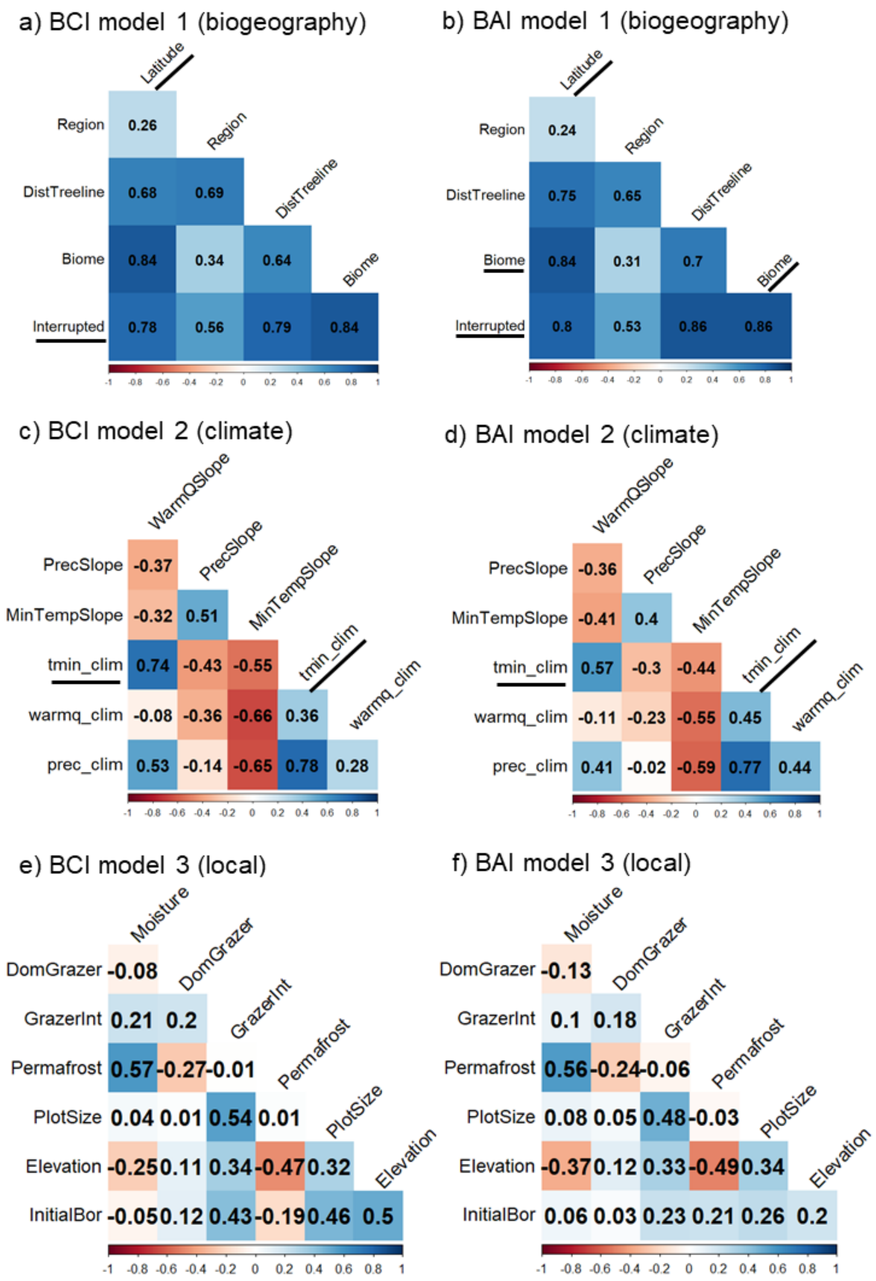

Figure S2. Correlograms displaying the pairwise correlations for the fixed effects in each community-level model. Colour intensity is proportional to the correlation coefficients, with blue and red tones indicating positive and negative correlations, respectively. Underlined variables indicate those that were removed in community models due to high correlation (|Spearman's r| > 0.7) with other variables (see Methods). In order to include categorical variables in the pairwise correlations, we coded them as ordinal variables as follows: moisture (dry = 0, moist = 1, mixed = 2, wet = 3), dominant grazer (none = 0, insects = 1, small mammals = 2, birds = 3, mixed = 4, large mammals = 5), grazer intensity (low = 1, medium = 2, high = 3), permafrost (none = 0, sporadic = 1, continuous = 2), interruption between site and treeline (uninterrupted = 0, small water bodies = 1, mountains = 2, large water bodies = 3), biome (alpine = 0, Arctic = 1), biogeographic region (Eurasia = 1, Western North America = 2, Eastern North America = 3, Greenland-Iceland = 4).

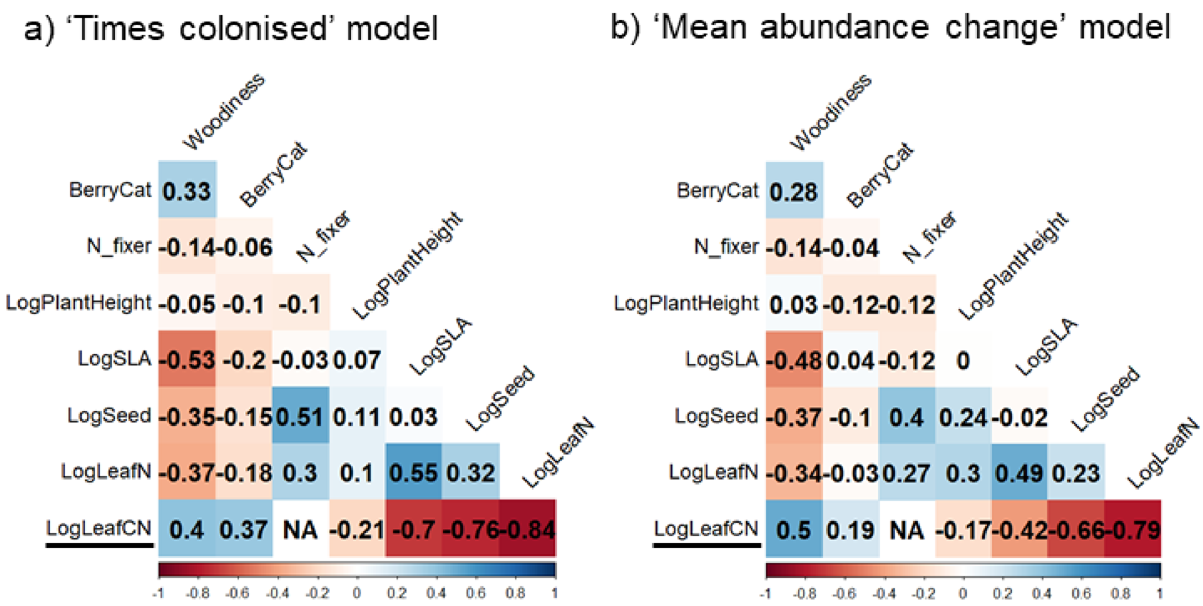

Figure S3. Correlogram with the pairwise correlations for the predictor variables in the species-level models. Colour intensity is proportional to the correlation coefficients, with blue and red tones indicating positive and negative correlations, respectively. The NA value between N_fixer and Leaf C:N is due to lack of variability in the data, since all species with Leaf C:N data were non-fixers (i.e., all had a 0 value). The underlined variable (leaf C:N) was removed in the species-level models due to high correlation with leaf N in both datasets, and with SLA and seed mass on the colonisation dataset (see Methods). Categorical traits were transformed to numerical variables for investigating correlations: berry production (not berry = 0, berry = 1), woodiness (not woody = 0, woody = 1), and N-fixer (0 = not fixer, 1 = fixer).

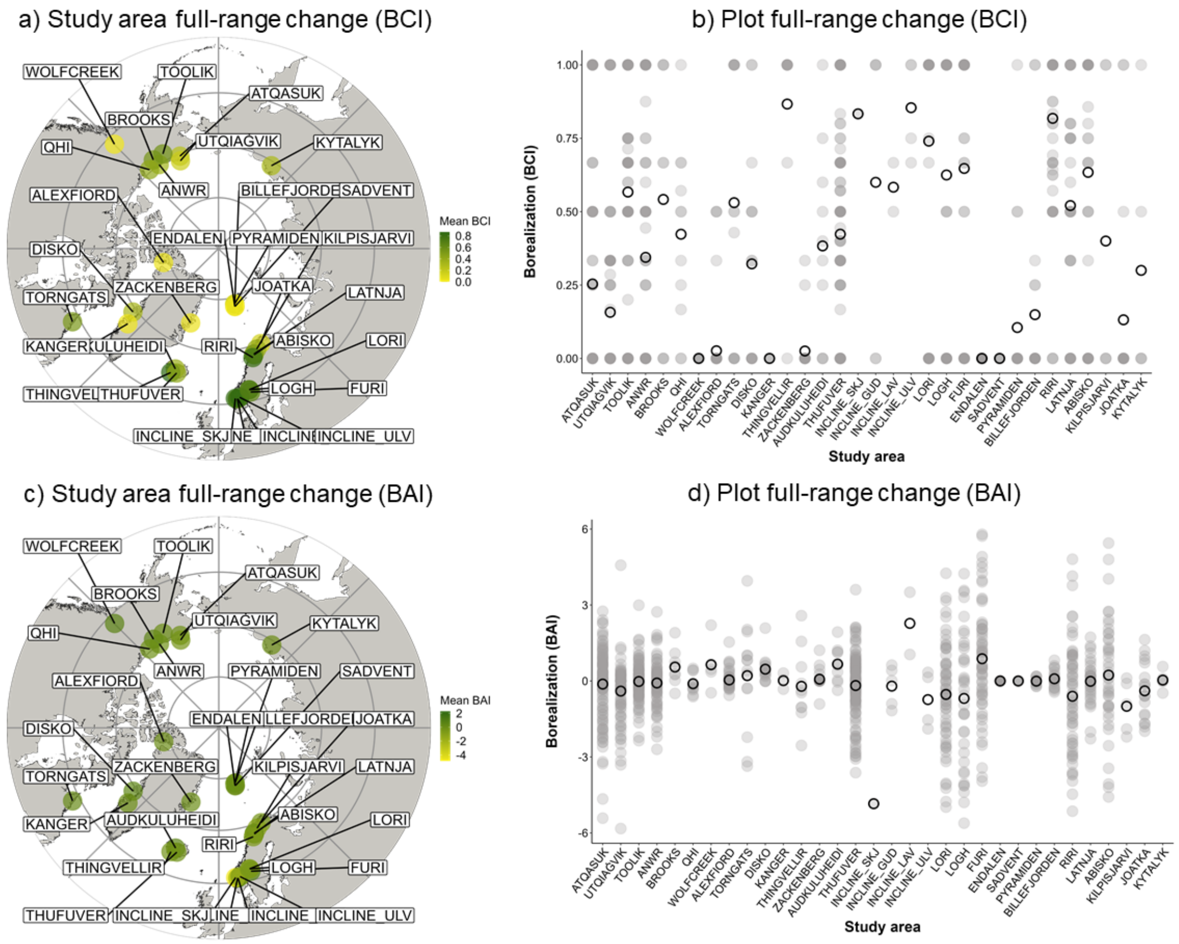

Figure S4. We detected wide variability in plant community borealisation rates across the tundra when including plots that did not experience boreal colonisations (BCI = 0) and plots that experienced either no change or decreases in boreal species abundance (BAI <=0). The panel shows the distribution of the full range of change for Boreal and Boreal-Tundra species across the tundra. **a)** Borealisation estimated using colonisations as an average of all plots within a study area, **b)** BCI index for each plot within each study area, **c)** Borealisation estimated using abundance change as an average of all plots within a study area, **d)** BAI index for each plot within each study area. Points in **a)** and **c)** are coloured according to the magnitude of increase (as BAI and BCI). Open circles indicate the mean value of the plot borealisation index at the study area level in **b)** and **d)**, which represent the same value as coloured points in **a)** and **c)**. Study areas in **b)** and **d)** are arranged by longitude, and darker grey colours indicate overlap of multiple points.

Supplementary Tables

Table S1. Classification of species according to their presence and frequency in the different boreal and Arctic zones. We include in each column all the frequency combinations for the species in our dataset that we assigned to each class. Other possible frequency combinations for species not included in our dataset are not represented here. The format in each row follows the following pattern: frequency in the Boreal zone ("Bor"), Zone E ("E"), Zone D ("D"), Zone C ("C"), Zone B ("B") and Zone A ("A"), as indicated in the top row (Walker et al. 2005). Frequency in the different zones is specified and colour-coded as follows: No = not present, X = present without a frequency assessment, ? = uncertain, * = stabilized introductions, ** = casual introductions, R = rare, b = borderline Arctic species only present in the southernmost part of Zone E, S = scattered and F = frequent. The frequency in each zone is extracted from the Arctic Biodiversity Assessment (Meltofte 2013), and in particular from Appendix 9.1 (Daniëls et al. 2013), which lists all Arctic vascular plant species and their distribution in the five subzones based on Elven (2007). We considered a species to be present in a particular zone when it was reported as 'scattered' or 'frequent' in Daniëls et al. (2013); in contrast, we considered a species to be absent in a particular zone if it was described as 'rare', 'uncertain' or 'introduced'.

Boreal

Bor	E	D	C	B	A
F	R	R	No	No	No
S	R	No	No	No	No
F	R	No	No	No	No
S	b	No	No	No	No
F	b	No	No	No	No
S	*	No	No	No	No
F	R	R	?	No	No

Boreal-Tundra

Bor	E	D	C	B	A
S	F	F	R	R	No
S	S	S	R	No	No
F	S	S	R	No	No
F	F	S	R	No	No
F	F	R	R	No	No
S	F	F	R	No	No
F	F	F	R	No	No
F	F	S	No	No	No
S	S	R	No	No	No
F	S	R	No	No	No
S	F	R	No	No	No
F	F	R	No	No	No
F	S	No	No	No	No
R	R	No	No	No	No
F	F	No	No	No	No
F	F	F	No	No	No
F	S	?	No	No	No
F	F	?	No	No	No
F	F	S	?	No	No
F	F	R	?	No	No
F	F	F	?	No	No
F	F	R	**	No	No
F	F	F	**	No	No
F	F	*	**	No	No
F	F	F	R	?	No

Arctic

Bor	E	D	C	B	A
R	R	S	F	F	S
R	S	F	F	F	R
R	S	S	S	R	No
R	F	F	F	R	No
R	F	F	S	No	No
R	F	S	R	No	No
R	F	F	R	No	No
No	X	X	No	No	No
R	S	F	F	F	F
No	S	F	F	F	F
No	R	F	F	F	F

Ubiquitous

Bor	E	D	C	B	A
F	F	S	F	F	S
S	F	F	F	F	S
F	F	F	F	F	S
S	F	F	F	S	R
S	F	F	F	F	R
F	F	F	F	F	R
S	F	F	F	S	No
F	F	F	F	S	No
S	F	F	S	R	No
F	F	F	S	R	No
S	F	F	F	R	No
F	F	F	F	R	No
S	F	F	S	No	No
F	F	F	S	No	No
F	F	F	F	No	No
F	F	F	S	?	No
F	F	F	F	?	No
S	F	F	F	F	F
F	F	F	F	F	F
S	F	F	F	S	?
F	F	F	F	S	?
F	F	F	F	R	?

Table S2. Model structure for community- and species-level models. Borealisation was quantified using BCI and BAI in community models, and as times colonised and mean cover change in the species-level models. Model structure is similar between 'BCI' and 'BAI' community models (*except for the BAI biogeographic model where biome was not included as a predictor), and between 'times colonised' and 'mean cover change' species models. Fixed effects are the retained variables after examining pairwise correlations among variables of interest. All community models included subsite as a random effect to account for spatial autocorrelation. Sample sizes are included in square brackets, first for the 'positive-only' models (i.e., including BCI and BAI values greater than zero) and then for 'full-range' models (i.e., including zeroes for BCI, and zeroes and negative values for BAI).

**Community-level models (Borealisation = BCI or BAI per plot)**
**Model 1: Biogeographic model** [*n* = BCI: 598, 1,137; BAI: 488, 1,137]<br>Borealisation ~ Biogeographic region + Distance to treeline + Biome* + (1|Subsite)
**Model 2: Climatic model** [*n* = BCI: 597, 1,130; BAI: 487, 1,130]<br>Borealisation ~ Warmest quarter temperature change + Precipitation change + Minimum Temperature change + Warmest quarter temperature climatology + Precipitation climatology + (1|Subsite)
**Model 3: Local model** [*n* = BCI: 581, 1,103; BAI: 466, 1,103]<br>Borealisation ~ Moisture + Grazing intensity + Dominant grazer + Elevation + Permafrost + Plot size + Initial boreal status + (1|Subsite)
**Species-level models (Borealisation = times colonised or mean cover per species)**<br>[*n* = 29 for times colonised model, *n* = 24 for abundance change model]
Borealisation ~ Functional group + log(Plant height) + log(SLA) + log(Seed mass) + log(Leaf N)

Table S3. Summary of the key Bayesian community-level and species-level models outlined in **Table S2**. CI means credible intervals. Parameters in bold indicate model estimates whose 95% credible intervals did not overlap zero (except for categorical variables).

Model number	Model name	Term	Estimate	Std. error	Lower 95% CI	Upper 95% CI
1	BAI Biogeographical (increases)	b_Intercept	1.143	0.136	0.87	1.413
		b_RegionGreenIceLand	-0.301	0.262	-0.81	0.229
		b_RegionNorthAmericaMEast	0.163	0.637	-1.09	1.467
		b_RegionNorthAmericaMWest	-0.573	0.167	-0.895	-0.241
		**b_DistanceTreelineCorrectedKmCentred**	**-0.001**	**0.0003**	**-0.001**	**-0.0001**
		sd_SiteSubsite__Intercept	0.438	0.079	0.291	0.601
		sigma	0.887	0.032	0.828	0.952
		lprior	-4.48	0.01	-4.502	-4.463
2	BAI Biogeographical (full range)	b_Intercept	-0.096	0.124	-0.338	0.144
		b_RegionGreenIceLand	0.138	0.275	-0.396	0.684
		b_RegionNorthAmericaMEast	0.039	0.519	-0.974	1.068
		b_RegionNorthAmericaMWest	0.079	0.184	-0.277	0.437
		b_DistanceTreelineCorrectedKmCentred	0.0001	0.0003	-0.0004	0.001
		sd_SiteSubsite__Intercept	0.587	0.072	0.454	0.736
		sigma	1.307	0.028	1.252	1.364
		lprior	-4.578	0.011	-4.601	-4.558
3	BAI Climate (increases)	b_Intercept	0.806	0.57	-0.344	1.895
		b_WarmQSlope	-6.838	4.964	-16.405	3.149
		**b_PrecSlope**	**-0.066**	**0.024**	**-0.112**	**-0.019**
		b_MinTempSlope	-1.859	2.897	-7.515	3.801
		b_warmq_clim	0.059	0.039	-0.017	0.138
		b_prec_clim	0.0003	0.0002	-0.00002	0.001
		sd_SiteSubsite__Intercept	0.348	0.092	0.164	0.526

		sigma	0.894	0.032	0.835	0.959
		lprior	-4.476	0.009	-4.497	-4.46
4	BAI Climate (full range)	b_Intercept	-0.811	0.607	-2.018	0.373
		b_WarmQSlope	9.402	6.301	-2.952	21.766
		b_PrecSlope	0.028	0.028	-0.029	0.084
		b_MinTempSlope	0.813	2.711	-4.628	6.103
		b_warmq_clim	0.048	0.045	-0.043	0.136
		b_prec_clim	-0.00002	0.0002	-0.0004	0.0005
		sd_SiteSubsite__Intercept	0.572	0.07	0.445	0.716
		sigma	1.306	0.029	1.253	1.364
		lprior	-4.576	0.011	-4.599	-4.556
5	BAI Local (increases)	b_Intercept	1.558	0.584	0.423	2.712
		b_MoistureMoist	-0.189	0.207	-0.577	0.23
		b_MoistureWet	-0.516	0.312	-1.105	0.118
		b_GrazerIntensityLow	-0.66	0.499	-1.635	0.314
		b_GrazerIntensityMedium	-0.373	0.496	-1.363	0.597
		b_DomGrazerInsects	0.256	0.43	-0.579	1.108
		b_DomGrazerLarge	0.229	0.319	-0.397	0.866
		b_DomGrazerMixed	-0.049	0.348	-0.738	0.626
		b_DomGrazerNone	0.726	0.488	-0.218	1.716
		b_DomGrazerSmall	0.367	0.346	-0.302	1.056
		b_ElevationSubsite	-0.0001	0.0003	-0.001	0.001
		b_permafrostnone	0.805	0.221	0.36	1.245
		b_permafrostsporadic	0.096	0.273	-0.435	0.632
		b_SurveyedArea	-0.002	0.003	-0.008	0.003
		**b_StartBorAbun**	**-0.011**	**0.002**	**-0.015**	**-0.007**
		sd_SiteSubsite__Intercept	0.413	0.092	0.238	0.596
		sigma	0.866	0.031	0.806	0.929
		lprior	-4.473	0.01	-4.495	-4.456

6	BAI Local (full range)	b_Intercept	1.272	0.74	-0.128	2.785
		b_MoistureMoist	0.413	0.234	-0.041	0.875
		b_MoistureWet	-0.184	0.369	-0.899	0.539
		b_GrazerIntensityLow	-1.082	0.599	-2.256	0.075
		b_GrazerIntensityMedium	-0.799	0.588	-1.974	0.342
		b_DomGrazerInsects	0.608	0.589	-0.567	1.771
		b_DomGrazerLarge	0.001	0.457	-0.921	0.871
		b_DomGrazerMixed	-0.459	0.53	-1.521	0.56
		b_DomGrazerNone	0.154	0.666	-1.17	1.459
		b_DomGrazerSmall	-0.145	0.478	-1.12	0.78
		**b_ElevationSubsite**	**0.001**	**0.0004**	**0.0002**	**0.002**
		b_permafrostnone	0.107	0.303	-0.484	0.704
		b_permafrostsporadic	-0.069	0.342	-0.744	0.612
		b_SurveyedArea	0.005	0.003	-0.0003	0.01
		**b_StartBorAbun**	**-0.027**	**0.002**	**-0.031**	**-0.024**
		sd_SiteSubsite__Intercept	0.749	0.089	0.59	0.935
		sigma	1.178	0.026	1.128	1.229
		lprior	-4.575	0.016	-4.611	-4.548
7	BCI Biogeographical (increases)	b_Intercept	1.784	0.197	1.401	2.182
		b_DistanceTreelineCorrectedKmCentred	-0.0004	0.0005	-0.001	0.001
		b_biomeArctic	-0.292	0.414	-1.118	0.491
		b_RegionGreenIceLand	-0.802	0.251	-1.29	-0.309
		b_RegionNorthAmericaMEast	-0.94	0.985	-2.9	0.982
		b_RegionNorthAmericaMWest	-0.194	0.35	-0.87	0.524
		sd_SiteSubsite__Intercept	0.367	0.094	0.194	0.557
		phi	1.237	0.082	1.084	1.406
		lprior	-8.257	0.082	-8.417	-8.098
8	BCI Biogeographical (full range)	b_Intercept	0.195	0.322	-0.419	0.833
		**b_DistanceTreelineCorrectedKmCentred**	**-0.004**	**0.001**	**-0.005**	**-0.002**

		b_biomeArctic	-0.124	0.629	-1.397	1.091
		b_RegionGreenIceLand	-0.355	0.469	-1.254	0.591
		b_RegionNorthAmericaMEast	1.515	1.235	-0.885	4.016
		b_RegionNorthAmericaMWest	-1.096	0.534	-2.14	-0.021
		sd_SiteSubsite__Intercept	0.935	0.134	0.698	1.223
		lprior	-3.24	0.028	-3.305	-3.196
9	BCI Climate (increases)	b_Intercept	0.999	0.599	-0.182	2.134
		**b_WarmQSlope**	**-15.776**	**4.517**	**-24.636**	**-6.843**
		**b_PrecSlope**	**-0.053**	**0.017**	**-0.086**	**-0.02**
		b_MinTempSlope	-1.327	3.378	-7.953	5.3
		**b_warmq_clim**	**0.177**	**0.044**	**0.092**	**0.263**
		b_prec_clim	-0.0002	0.0002	-0.001	0.0002
		sd_SiteSubsite__Intercept	0.105	0.072	0.005	0.269
		phi	1.253	0.08	1.101	1.415
		lprior	-8.275	0.08	-8.429	-8.115
10	BCI Climate (full range)	b_Intercept	-3.805	1.09	-6.019	-1.772
		b_WarmQSlope	-8.391	9.723	-27.808	10.807
		b_PrecSlope	-0.021	0.048	-0.114	0.076
		b_MinTempSlope	3.786	5.209	-6.178	14.206
		**b_warmq_clim**	**0.397**	**0.078**	**0.248**	**0.55**
		**b_prec_clim**	**0.001**	**0.0004**	**0.001**	**0.002**
		sd_SiteSubsite__Intercept	0.912	0.149	0.648	1.228
		lprior	-3.233	0.029	-3.299	-3.187
11	BCI Local (increases)	b_Intercept	1.889	0.671	0.61	3.215
		b_MoistureMoist	-0.08	0.205	-0.482	0.328
		b_MoistureWet	-0.486	0.311	-1.094	0.139
		b_GrazerIntensityLow	-0.974	0.594	-2.188	0.128
		b_GrazerIntensityMedium	-0.83	0.594	-2.023	0.278
		b_DomGrazerInsects	-0.621	0.525	-1.666	0.423

		b_DomGrazerLarge	0.375	0.309	-0.27	0.977
		b_DomGrazerMixed	0.389	0.334	-0.273	1.059
		b_DomGrazerNone	0.899	0.425	0.084	1.766
		b_DomGrazerSmall	0.532	0.338	-0.167	1.182
		b_ElevationSubsite	0.0003	0.0003	-0.0003	0.001
		b_permafrostnone	0.419	0.234	-0.032	0.89
		b_permafrostsporadic	-0.173	0.296	-0.742	0.434
		b_SurveyedArea	-0.006	0.003	-0.012	0.001
		b_StartBorSpps	0.0003	0.021	-0.04	0.041
		sd_SiteSubsite__Intercept	0.29	0.122	0.052	0.532
		phi	1.258	0.085	1.098	1.43
		lprior	-8.283	0.086	-8.45	-8.115
12	BCI Local (full range)	b_Intercept	-1.136	1.214	-3.504	1.316
		b_MoistureMoist	0.459	0.411	-0.337	1.272
		b_MoistureWet	0.287	0.621	-0.915	1.532
		b_GrazerIntensityLow	-1.226	0.944	-3.068	0.571
		b_GrazerIntensityMedium	-0.467	0.935	-2.32	1.35
		b_DomGrazerInsects	-0.82	0.973	-2.764	1.024
		b_DomGrazerLarge	-0.629	0.764	-2.181	0.83
		b_DomGrazerMixed	-0.001	0.89	-1.758	1.71
		b_DomGrazerNone	0.583	1.14	-1.692	2.785
		b_DomGrazerSmall	-0.21	0.809	-1.891	1.294
		**b_ElevationSubsite**	**0.002**	**0.001**	**0.001**	**0.003**
		b_permafrostnone	2.464	0.532	1.447	3.552
		b_permafrostsporadic	1.239	0.646	-0.032	2.511
		**b_SurveyedArea**	**0.014**	**0.005**	**0.005**	**0.023**
		b_StartBorSpps	-0.043	0.035	-0.113	0.025
		sd_SiteSubsite__Intercept	1.198	0.19	0.869	1.614
		lprior	-3.301	0.05	-3.419	-3.224

13	Species abundance change per class	b_Intercept	0.269	0.069	0.131	0.399
		b_ClassNewBorealMtundraboundary	-0.083	0.072	-0.219	0.062
		b_ClassNewBorealspecialist	0.01	0.106	-0.198	0.224
		b_ClassNewUbiquitous	-0.098	0.075	-0.243	0.056
		sigma	0.184	0.012	0.164	0.209
		lprior	-3.146	0.001	-3.147	-3.145
14	Species abundance change (multivariate)	b_Intercept	2.058	0.676	0.711	3.401
		b_FunctionalGroupGraminoid	-0.224	0.109	-0.441	-0.01
		b_FunctionalGroupShrub	-0.323	0.137	-0.595	-0.05
		b_logPlantHeight	0.074	0.062	-0.05	0.199
		b_logSLA	-0.2	0.123	-0.44	0.048
		b_logSeedMass	-0.014	0.031	-0.075	0.047
		b_logLeafN	-0.349	0.178	-0.702	0.004
		sigma	0.184	0.033	0.132	0.264
		lprior	-3.146	0.002	-3.15	-3.144
15	Species abundance change vs functional group (univariate)	b_Intercept	0.194	0.029	0.137	0.252
		b_FunctionalGroupGraminoid	-0.005	0.059	-0.12	0.113
		b_FunctionalGroupShrub	-0.002	0.054	-0.111	0.102
		sigma	0.201	0.016	0.171	0.235
		lprior	-3.146	0.001	-3.148	-3.145
16	Species abundance change vs height (univariate)	b_Intercept	0.236	0.057	0.122	0.352
		b_logPlantHeight	0.023	0.029	-0.033	0.08
		sigma	0.21	0.021	0.174	0.254
		lprior	-3.147	0.001	-3.15	-3.145
17	Species abundance change vs SLA (univariate)	b_Intercept	0.365	0.17	0.033	0.699
		b_logSLA	-0.07	0.059	-0.188	0.046
		sigma	0.183	0.019	0.15	0.227
		lprior	-3.145	0.001	-3.147	-3.144
18		b_Intercept	0.201	0.046	0.11	0.29

	Species abundance change vs seed mass (univariate)	b_logSeedMass	0.008	0.026	-0.042	0.058
		sigma	0.211	0.031	0.16	0.281
		lprior	-3.147	0.002	-3.151	-3.145
19	Species abundance change vs leaf N (univariate)	b_Intercept	0.274	0.241	-0.195	0.746
		b_logLeafN	-0.03	0.08	-0.187	0.128
		sigma	0.193	0.022	0.155	0.241
		lprior	-3.145	0.001	-3.148	-3.144
20	Times colonised per class	b_Intercept	2.046	0.382	1.34	2.831
		b_ClassNewBorealMtundraboundary	0.176	0.393	-0.638	0.915
		b_ClassNewBorealspecialist	-0.86	0.555	-1.945	0.242
		b_ClassNewUbiquitous	0.277	0.4	-0.541	1.02
		shape	0.925	0.09	0.759	1.109
		lprior	-6.53	0.098	-6.717	-6.335
21	Times colonised (multivariate)	b_Intercept	-1.327	2.5	-6.199	3.684
		b_FunctionalGroupGraminoid	1.147	0.481	0.183	2.09
		b_FunctionalGroupShrub	1.961	0.647	0.703	3.248
		**b_logPlantHeight**	**-0.808**	**0.22**	**-1.243**	**-0.36**
		b_logSLA	0.845	0.656	-0.493	2.143
		b_logSeedMass	0.091	0.162	-0.224	0.41
		b_logLeafN	-0.251	0.684	-1.623	1.064
		shape	1.876	0.634	0.915	3.347
		lprior	-7.157	0.331	-7.796	-6.496
22	Times colonised vs functional group (univariate)	b_Intercept	1.926	0.142	1.657	2.209
		b_FunctionalGroupGraminoid	0.1	0.249	-0.372	0.591
		b_FunctionalGroupShrub	0.756	0.252	0.277	1.259
		shape	0.928	0.119	0.713	1.176
		lprior	-6.546	0.128	-6.791	-6.291
23	Times colonised vs height (univariate)	b_Intercept	1.686	0.25	1.21	2.2
		**b_logPlantHeight**	**-0.322**	**0.112**	**-0.543**	**-0.099**

		shape	0.984	0.15	0.719	1.305
		lprior	-6.599	0.153	-6.896	-6.29
24	Times colonised vs SLA (univariate)	b_Intercept	4.391	0.731	2.963	5.85
		**b_logSLA**	**-0.718**	**0.257**	**-1.217**	**-0.211**
		shape	1.041	0.168	0.744	1.403
		lprior	-6.636	0.161	-6.948	-6.314
25	Times colonised vs seed mass (univariate)	b_Intercept	2.547	0.212	2.154	2.987
		b_logSeedMass	-0.139	0.135	-0.403	0.126
		shape	1.009	0.232	0.629	1.523
		lprior	-6.593	0.227	-7.031	-6.148
26	Times colonised vs leaf N (univariate)	b_Intercept	3.586	1.11	1.46	5.826
		b_logLeafN	-0.336	0.364	-1.069	0.37
		shape	0.974	0.173	0.678	1.343
		lprior	-6.557	0.177	-6.894	-6.212

Table S4. Summary of change and relevant traits per species, ordered by the number of times it colonised different plots. Plant species classes (Boreal [B], Boreal-Tundra [BT], Arctic [A] and Ubiquitous [U]) are described in **Table S1**. 95% confidence intervals are calculated per species as (standard deviation/√n)*1.96. Species with NA values of mean plant height did not have enough records (minimum of 5) to be retained for species-level analysis. Sample size refers to the number of plots where the species were recorded in.

Order	Species	Functional group	Class	Times colonised	Mean cover change (% per year)	Cover change confidence intervals	Mean plant height (m)	Sample size
1	*Empetrum nigrum*	Shrub	U	72	0.27	0.16	0.10	469
2	*Persicaria vivipara*	Forb	U	67	0.08	0.12	0.09	345
3	*Carex bigelowii*	Graminoid	BT	62	-0.08	0.07	0.09	536
4	*Betula nana*	Shrub	BT	56	0.04	0.13	0.19	381
5	*Phyllodoce caerulea*	Shrub	BT	49	0.11	0.18	0.07	123
6	*Vaccinium vitis-idaea*	Shrub	BT	48	0.00	0.06	0.05	559
7	*Vaccinium myrtillus*	Shrub	BT	43	0.02	0.17	0.09	219
8	*Silene acaulis*	Forb	U	42	-0.38	0.20	0.01	155
9	*Harrimanella hypnoides*	Shrub	BT	41	-0.13	0.17	0.01	110
10	*Persicaria bistorta*	Forb	BT	38	-0.03	0.06	NA	143
11	*Arctagrostis latifolia*	Graminoid	U	36	0.00	0.08	0.26	134

12	*Poa arctica*	Graminoid	U	36	0.39	0.21	0.14	137
13	*Stellaria longipes*	Forb	U	33	-0.10	0.11	0.06	112
14	*Salix arctica*	Shrub	U	31	0.23	0.16	0.04	236
15	*Pinguicula vulgaris*	Forb	BT	30	0.09	0.04	0.08	37
16	*Pedicularis lapponica*	Forb	BT	29	-0.05	0.08	0.07	61
17	*Thalictrum alpinum*	Forb	BT	29	-0.07	0.12	0.06	78
18	*Cassiope tetragona*	Shrub	U	27	-0.17	0.10	0.09	291
19	*Rubus chamaemorus*	Forb	BT	27	-0.03	0.09	0.06	117
20	*Petasites frigidus*	Forb	U	26	0.13	0.14	0.15	89
21	*Salix daphnoides*	Shrub	BT	26	-0.26	0.15	0.35	163
22	*Salix lanata*	Shrub	BT	25	0.28	0.20	1.48	32
23	*Eriophorum angustifolium*	Graminoid	U	24	0.38	0.21	0.25	143
24	*Vaccinium uliginosum*	Shrub	U	23	0.07	0.10	0.11	188
25	*Eriophorum vaginatum*	Graminoid	BT	23	0.38	0.13	0.18	222
26	*Tofieldia pusilla*	Forb	BT	22	0.00	0.08	0.11	56
27	*Deschampsia flexuosa*	Graminoid	BT	19	-0.15	0.14	0.12	200

28	*Luzula confusa*	Graminoid	U	19	-0.22	0.09	0.12	149
29	*Luzula nivalis*	Graminoid	A	19	-0.17	0.14	0.10	96
30	*Pyrola grandiflora*	Forb	U	19	-0.02	0.07	0.05	49
31	*Kobresia myosuroides*	Graminoid	U	19	0.11	0.17	NA	60
32	*Saxifraga cernua*	Forb	U	18	-0.08	0.16	0.10	60
33	*Saxifraga hirculus*	Forb	U	18	-0.01	0.15	0.18	52
34	*Calluna vulgaris*	Shrub	BT	18	0.42	0.41	0.17	105
35	*Micranthes punctata*	Forb	BT	17	0.00	0.09	NA	44
36	*Tephroseris integrifolia*	Forb	BT	17	-0.03	0.06	0.15	51
37	*Dupontia fisheri*	Graminoid	U	17	-0.27	0.39	0.17	83
38	*Saxifraga oppositifolia*	Forb	U	16	-0.14	0.29	NA	65
39	*Trisetum spicatum*	Graminoid	U	16	0.29	0.19	0.24	46
40	*Luzula spicata*	Graminoid	BT	15	0.06	0.07	0.13	25
41	*Carex microcarpa*	Graminoid	U	15	0.43	0.26	0.22	145
42	*Rhododendron tomentosum*	Shrub	BT	15	-0.21	0.10	0.08	235
43	*Salix polaris*	Shrub	A	15	-0.05	0.32	0.04	117

44	*Solidago virgaurea*	Forb	BT	14	0.15	0.12	0.12	74
45	*Oxyria digyna*	Forb	U	14	-0.10	0.08	0.17	80
46	*Salix herbacea*	Shrub	U	14	-0.14	0.17	0.02	214
47	*Ranunculus nivalis*	Forb	U	14	-0.09	0.11	0.10	27
48	*Minuartia stricta*	Forb	BT	14	0.17	0.10	NA	26
49	*Bartsia alpina*	Forb	BT	13	0.11	0.08	0.08	21
50	*Andromeda polifolia*	Shrub	BT	13	0.01	0.17	0.05	60
51	*Pedicularis hirsuta*	Forb	U	13	0.03	0.10	0.08	21
52	*Hierochloe pauciflora*	Graminoid	U	13	0.43	0.42	0.13	27
53	*Salix glauca*	Shrub	BT	12	0.22	0.20	0.46	47
54	*Oreojuncus trifidus*	Graminoid	BT	12	0.01	0.06	0.09	43
55	*Festuca richardsonii*	Graminoid	U	12	0.00	0.29	0.31	80
56	*Arctous alpina*	Shrub	U	11	-0.02	0.16	0.05	43
57	*Saussurea alpina*	Forb	BT	11	0.39	0.42	0.17	13
58	*Pedicularis capitata*	Forb	U	11	-0.03	0.07	0.08	31
59	*Hierochloe alpina*	Graminoid	U	11	0.12	0.14	0.24	50

60	*Astragalus alpinus*	Forb	BT	10	0.23	0.26	0.08	25
61	*Juniperus communis*	Shrub	BT	10	-0.77	0.85	0.60	23
62	*Juncus biglumis*	Graminoid	U	10	0.10	0.11	0.08	20
63	*Salix rotundifolia*	Shrub	BT	10	-0.63	0.28	0.02	53
64	*Saussurea angustifolia*	Forb	BT	10	0.01	0.06	0.13	24
65	*Armeria maritima*	Forb	BT	10	-0.36	0.10	0.19	100
66	*Sagina nivalis*	Forb	A	10	0.31	0.05	0.02	10
67	*Viola biflora*	Forb	BT	10	0.18	0.15	0.04	32
68	*Salix reticulata*	Shrub	U	9	0.32	0.16	0.05	111
69	*Lysimachia europaea*	Forb	BT	9	-0.03	0.03	0.04	77
70	*Diapensia lapponica*	Shrub	U	9	-0.03	0.12	0.03	40
71	*Sibbaldia procumbens*	Forb	BT	9	-0.01	0.16	0.03	24
72	*Agrostis mertensii*	Graminoid	BT	9	-0.29	0.17	0.14	39
73	*Pedicularis flammea*	Forb	A	9	0.05	0.10	NA	16
74	*Carex rupestris*	Graminoid	U	8	-0.06	0.14	NA	30
75	*Calamagrostis lapponica*	Graminoid	BT	8	-0.08	0.19	0.19	91

76	*Deschampsia cespitosa*	Graminoid	BT	8	-0.08	0.29	0.60	22
77	*Dryas integrifolia*	Shrub	U	8	-0.12	0.10	0.06	125
78	*Pedicularis lanata*	Forb	U	8	0.03	0.14	0.08	28
79	*Anthoxanthum odoratum*	Graminoid	B	8	-0.43	0.57	0.26	19
80	*Festuca ovina*	Graminoid	BT	7	0.05	0.30	0.35	36
81	*Carex fuliginosa*	Graminoid	U	7	-0.06	0.09	NA	24
82	*Festuca rubra*	Graminoid	U	7	0.11	0.36	0.31	25
83	*Potentilla hyparctica*	Forb	U	7	-0.10	0.09	0.07	26
84	*Micranthes foliolosa*	Forb	U	7	-0.26	0.22	0.09	22
85	*Luzula multiflora*	Graminoid	BT	7	0.01	0.03	0.15	45
86	*Rhodiola rosea*	Forb	U	7	0.08	0.20	0.11	15
87	*Eriophorum scheuchzeri*	Graminoid	U	6	0.21	0.14	0.24	6
88	*Salix phlebophylla*	Shrub	BT	6	0.06	0.26	0.02	54
89	*Alopecurus alpinus*	Graminoid	U	6	-0.11	0.44	0.21	24
90	*Cochlearia officinalis*	Forb	BT	6	-0.03	0.13	0.15	14
91	*Poa pratensis*	Graminoid	U	6	-0.16	0.10	0.28	29

92	*Luzula arcuata*	Graminoid	BT	6	-0.02	0.09	NA	11
93	*Galium boreale*	Forb	BT	6	0.75	0.42	0.28	6
94	*Dryas octopetala*	Shrub	U	5	0.03	0.28	0.05	89
95	*Rhododendron lapponicum*	Shrub	U	5	-0.01	0.33	0.08	29
96	*Cardamine bellidifolia*	Forb	U	5	0.20	0.18	NA	6
97	*Oreomecon radicata*	Forb	B	5	-0.08	0.08	0.11	22
98	*Cerastium alpinum*	Forb	U	5	-0.04	0.08	NA	15
99	*Oxytropis nigrescens*	Forb	U	5	0.04	0.13	NA	19
100	*Pedicularis sudetica*	Forb	U	5	-0.14	0.21	0.08	13
101	*Carex rotundata*	Graminoid	BT	5	0.08	0.82	NA	10
102	*Carex capillaris*	Graminoid	BT	5	-0.05	0.12	NA	12
103	*Cardamine pratensis*	Forb	U	5	-0.30	0.17	0.18	25
104	*Cerastium arcticum*	Forb	A	5	0.03	0.14	0.11	20
105	*Carex microglochin*	Graminoid	BT	5	0.14	0.10	NA	5
106	*Ranunculus acris*	Forb	BT	5	0.11	0.12	0.24	14
107	*Kalmia procumbens*	Shrub	BT	5	0.42	0.62	0.04	13

108	*Cardamine digitalis*	Forb	BT	5	0.05	0.05	0.05	6
109	*Gnaphalium supinum*	Forb	BT	5	-0.04	0.29	0.05	18
110	*Vahlodea atropurpurea*	Graminoid	BT	5	0.02	0.04	NA	11
111	*Euphrasia frigida*	Forb	A	5	-0.05	0.08	0.08	12
112	*Minuartia biflora*	Forb	U	5	0.07	0.02	NA	5
113	*Calamagrostis inexpansa*	Graminoid	B	5	-0.09	0.15	NA	25
114	*Poa glauca*	Graminoid	U	4	-0.27	0.42	0.26	8
115	*Calamagrostis stricta*	Graminoid	U	4	0.29	0.42	0.15	13
116	*Oxytropis maydelliana*	Forb	U	4	-0.09	0.14	0.09	16
117	*Oxytropis campestris*	Forb	BT	4	0.08	0.24	0.11	9
118	*Carex rariflora*	Graminoid	BT	4	0.45	0.84	0.28	13
119	*Festuca vivipara*	Graminoid	BT	4	0.17	0.30	0.14	8
120	*Cerastium beeringianum*	Forb	U	4	-0.46	0.37	NA	19
121	*Festuca altaica*	Graminoid	BT	4	0.39	0.15	NA	7
122	*Alopecurus magellanicus*	Graminoid	U	4	0.50	0.86	0.13	12
123	*Betula pubescens*	Shrub	BT	4	0.11	0.11	2.34	5

124	*Hieracium alpinum*	Forb	BT	4	-0.04	0.04	0.13	76
125	*Galium verum*	Forb	BT	4	0.83	0.78	0.15	5
126	*Salix hastata*	Shrub	BT	3	0.28	0.40	NA	9
127	*Salix chamissonis*	Shrub	BT	3	-0.06	0.26	NA	11
128	*Linnaea borealis*	Shrub	BT	3	0.02	0.50	0.05	6
129	*Festuca brachyphylla*	Graminoid	U	3	-0.20	0.29	0.12	11
130	*Arnica griscomii*	Forb	BT	3	0.19	0.54	NA	4
131	*Hedysarum alpinum*	Forb	BT	3	0.07	0.10	NA	12
132	*Antennaria friesiana*	Forb	U	3	0.11	0.25	0.10	4
133	*Draba lactea*	Forb	U	3	0.02	0.23	0.05	11
134	*Calamagrostis holmii*	Graminoid	BT	3	0.27	0.03	0.13	3
135	*Micranthes hieraciifolia*	Forb	U	3	0.03	0.12	NA	16
136	*Stellaria crassipes*	Forb	U	3	0.23	0.23	0.06	4
137	*Veronica alpina*	Forb	U	3	-0.01	0.09	0.07	15
138	*Pyrola minor*	Forb	BT	3	0.24	0.36	0.10	9
139	*Rumex acetosa*	Forb	BT	3	-0.04	0.07	0.32	18

140	*Gentiana nivalis*	Forb	BT	3	0.08	0.14	0.08	4
141	*Antennaria alpina*	Forb	BT	3	0.05	0.22	0.11	5
142	*Festuca baffinensis*	Graminoid	U	3	-0.09	0.23	0.15	6
143	*Euphrasia arctica*	Forb	B	3	0.37	0.25	NA	4
144	*Chamorchis alpina*	Forb	B	2	-0.03	0.26	NA	3
145	*Gymnadenia conopsea*	Forb	BT	2	0.17	0.07	0.40	2
146	*Pinguicula villosa*	Forb	BT	2	0.03	0.03	NA	5
147	*Orthilia secunda*	Forb	BT	2	-0.05	0.05	0.11	12
148	*Braya purpurascens*	Forb	U	2	-0.04	0.14	NA	6
149	*Salix planifolia*	Shrub	BT	2	0.01	0.30	0.44	44
150	*Astragalus umbellatus*	Forb	BT	2	-0.47	0.28	0.09	21
151	*Carex x turfosa*	Graminoid	BT	2	-0.05	0.06	0.19	6
152	*Anemone parviflora*	Forb	BT	2	0.10	0.22	NA	8
153	*Arctous rubra*	Shrub	BT	2	-0.01	0.20	NA	13
154	*Lupinus arcticus*	Forb	BT	2	0.54	0.41	0.17	16
155	*Pedicularis verticillata*	Forb	BT	2	-0.02	0.09	0.14	7

156	*Salix fuscescens*	Shrub	BT	2	0.15	0.99	NA	6
157	*Valeriana capitata*	Forb	BT	2	-0.06	0.10	0.19	6
158	*Ranunculus pallasii*	Forb	BT	2	0.07	0.35	NA	3
159	*Poa alpina*	Graminoid	U	2	-0.14	0.11	0.19	19
160	*Silene uralensis*	Forb	U	2	0.15	0.05	0.04	2
161	*Carex fimbriata*	Graminoid	B	2	-0.16	0.21	0.22	9
162	*Pedicularis dasyantha*	Forb	A	2	0.10	0.21	NA	6
163	*Silene apetala*	Forb	U	2	0.16	0.37	NA	4
164	*Phleum alpinum*	Graminoid	BT	2	-0.07	0.09	0.27	12
165	*Pyrola rotundifolia*	Forb	BT	2	0.32	0.37	0.23	3
166	*Carex lachenalii*	Graminoid	U	2	-0.40	0.16	0.11	46
167	*Carex brunnescens*	Graminoid	BT	2	-0.60	0.32	0.11	23
168	*Salix phylicifolia*	Shrub	BT	2	0.20	0.08	2.69	2
169	*Carex parallela*	Graminoid	U	1	-0.59	0.52	NA	19
170	*Carex vaginata*	Graminoid	BT	1	-0.43	0.24	0.09	45
171	*Astragalus frigidus*	Forb	BT	1	-0.16	0.21	NA	4

172	*Vaccinium microcarpum*	Shrub	BT	1	0.32	0.31	NA	6
173	*Carex ericetorum*	Graminoid	B	1	0.36	0.24	0.22	15
174	*Kobresia simpliciuscula*	Graminoid	U	1	0.18	NA	NA	1
175	*Silene involucrata*	Forb	U	1	0.07	NA	NA	1
176	*Androsace ochotensis*	Forb	A	1	0.50	NA	NA	1
177	*Eutrema edwardsii*	Forb	U	1	-0.20	0.18	NA	8
178	*Oxytropis borealis*	Forb	BT	1	-0.11	0.16	NA	11
179	*Salix niphoclada*	Shrub	BT	1	0.04	0.22	0.44	5
180	*Hedysarum boreale*	Forb	BT	1	0.05	0.15	NA	2
181	*Pedicularis labradorica*	Forb	BT	1	0.09	0.26	NA	2
182	*Minuartia arctica*	Forb	U	1	-0.12	0.42	NA	2
183	*Carex chordorrhiza*	Graminoid	BT	1	0.26	NA	NA	1
184	*Luzula wahlenbergii*	Graminoid	U	1	-0.04	0.20	0.20	2
185	*Papaver lapponicum*	Forb	U	1	-0.15	0.18	0.13	5
186	*Carex subspathacea*	Graminoid	U	1	-1.13	1.35	0.09	7
187	*Arctophila fulva*	Graminoid	U	1	-0.07	0.39	NA	3

188	*Ranunculus pygmaeus*	Forb	U	1	-0.63	1.78	0.05	2
189	*Cerastium regelii*	Forb	A	1	0.71	NA	NA	1
190	*Carex mackenziei*	Graminoid	BT	1	-0.09	0.23	NA	2
191	*Saxifraga tricuspidata*	Forb	U	1	-0.11	0.26	NA	2
192	*Draba nivalis*	Forb	U	1	-0.58	0.48	NA	6
193	*Aconitum septentrionale*	Forb	BT	1	0.54	NA	1.00	1
194	*Agrostis capillaris*	Graminoid	BT	1	-0.02	1.34	0.39	10
195	*Euphrasia wettsteinii*	Forb	U	1	0.06	0.13	NA	7
196	*Potentilla erecta*	Forb	B	1	0.28	0.61	0.20	4
197	*Scorzoneroides autumnalis*	Forb	BT	1	0.16	0.23	0.16	9
198	*Viola palustris*	Forb	BT	1	0.29	0.63	0.12	7
199	*Dactylorhiza viridis*	Forb	BT	1	0.00	0.31	0.22	2
200	*Parnassia palustris*	Forb	BT	1	0.03	0.09	0.13	3
201	*Potentilla crantzii*	Forb	U	1	-0.07	0.23	0.13	9
202	*Cerastium cerastoides*	Forb	BT	1	0.05	0.20	0.04	5
203	*Trollius europaeus*	Forb	BT	1	0.03	0.21	0.50	2

204	*Taraxacum croceum*	Forb	BT	1	0.01	0.10	NA	2
205	*Rumex alpestris*	Forb	BT	1	0.07	NA	0.32	1
206	*Micranthes nelsoniana*	Forb	U	1	-0.02	0.10	0.15	2
207	*Lagotis minor*	Forb	U	1	0.02	NA	0.14	1
208	*Galium normanii*	Forb	B	1	0.35	NA	NA	1
209	*Avenella flexuosa*	Graminoid	BT	1	-0.17	0.99	NA	4
210	*Koenigia islandica*	Forb	U	1	0.43	NA	0.04	1
211	*Thymus praecox*	Shrub	BT	1	0.09	NA	0.09	1
212	*Arnica lessingii*	Forb	BT	1	0.12	NA	NA	1
213	*Lagotis glauca*	Forb	U	1	-0.05	0.19	0.14	3
214	*Polemonium acutiflorum*	Forb	BT	1	0.08	NA	0.40	1
215	*Geum glaciale*	Forb	U	1	0.09	NA	NA	1
216	*Ranunculus lapponicus*	Forb	U	1	-0.16	0.87	NA	2
217	*Epilobium latifolium*	Forb	U	1	0.16	0.23	0.16	2
218	*Salix uva-ursi*	Shrub	BT	1	-0.25	0.91	NA	3
219	*Salix arctophila*	Shrub	BT	1	0.87	1.49	0.09	2

220	*Carex supina*	Graminoid	BT	1	0.34	0.88	NA	4
221	*Pseudorchis albida*	Forb	B	0	-0.14	NA	0.22	1
222	*Carex atrata*	Graminoid	BT	0	-0.08	0.06	0.22	7
223	*Carex marina*	Graminoid	A	0	-0.27	0.16	NA	7
224	*Carex microchaeta*	Graminoid	BT	0	-0.20	0.32	NA	8
225	*Artemisia norvegica*	Forb	B	0	0.05	0.16	NA	6
226	*Boykinia richardsonii*	Forb	BT	0	0.77	NA	NA	1
227	*Salix alaxensis*	Shrub	BT	0	-0.30	0.33	1.65	8
228	*Astragalus eucosmus*	Forb	BT	0	-0.04	0.02	NA	2
229	*Cardamine digitata*	Forb	BT	0	-0.36	0.52	0.05	3
230	*Doronicum grandiflorum*	Forb	B	0	-0.09	0.05	NA	2
231	*Senecio lugens*	Forb	BT	0	-0.03	NA	NA	1
232	*Eriophorum chamissonis*	Graminoid	BT	0	-0.09	0.14	0.18	3
233	*Oxytropis deflexa*	Forb	BT	0	-0.16	NA	NA	1
234	*Carex scirpoidea*	Graminoid	BT	0	0.02	NA	0.12	1
235	*Kobresia sibirica*	Graminoid	A	0	-0.08	NA	NA	1

236	*Minuartia obtusiloba*	Forb	BT	0	-0.05	0.30	NA	2
237	*Draba micropetala*	Forb	A	0	-0.13	0.11	0.04	2
238	*Stellaria humifusa*	Forb	U	0	-0.28	0.08	NA	5
239	*Chrysosplenium tetrandrum*	Forb	U	0	-0.31	0.13	0.02	2
240	*Gentianella propinqua*	Forb	BT	0	-0.20	0.03	0.10	2
241	*Potentilla fruticosa*	Shrub	BT	0	-0.05	0.08	0.48	2
242	*Salix arbusculoides*	Shrub	BT	0	-0.10	NA	0.27	1
243	*Saxifraga flagellaris*	Forb	A	0	-0.09	NA	NA	1
244	*Luzula parviflora*	Graminoid	BT	0	-0.04	NA	NA	1
245	*Saxifraga cespitosa*	Forb	U	0	-0.47	0.25	NA	2
246	*Achillea millefolium*	Forb	BT	0	0.32	0.41	0.15	3
247	*Alchemilla alpina*	Forb	BT	0	0.00	0.66	0.11	9
248	*Campanula rotundifolia*	Forb	BT	0	-0.08	0.15	0.25	6
249	*Carex flava*	Graminoid	B	0	-0.16	0.04	0.49	2
250	*Cerastium fontanum*	Forb	BT	0	-0.20	0.29	0.25	4
251	*Geranium sylvaticum*	Forb	BT	0	0.22	0.68	0.41	3

252	*Nardus stricta*	Graminoid	BT	0	-1.48	2.05	0.21	6
253	*Rhinanthus minor*	Forb	BT	0	0.04	0.29	0.35	3
254	*Sagina saginoides*	Forb	BT	0	-0.20	0.03	0.07	2
255	*Omalotheca supina*	Forb	BT	0	-0.54	0.68	NA	4
256	*Pilosella officinarum*	Forb	B	0	-0.35	1.61	0.08	2
257	*Epilobium anagallidifolium*	Forb	BT	0	-0.14	0.17	0.07	2
258	*Antennaria pulchella*	Forb	A	0	0.18	NA	NA	1
259	*Saxifraga aizoides*	Forb	U	0	-0.06	NA	0.08	1
260	*Lycopodium complanatum*	Forb	B	0	-0.01	0.00	0.09	2
261	*Erigeron humilis*	Forb	U	0	-0.31	0.28	NA	9
262	*Antennaria dioica*	Forb	BT	0	-0.03	NA	0.10	1
263	*Agrostis vinealis*	Graminoid	BT	0	-1.39	0.00	0.49	2
264	*Micranthes nivalis*	Forb	U	0	-0.16	NA	0.09	1
265	*Arabidopsis lyrata*	Forb	U	0	-0.06	NA	NA	1
266	*Androsace chamaejasme*	Forb	BT	0	-0.27	NA	NA	1
267	*Betula glandulosa*	Shrub	BT	0	0.16	1.67	0.56	5

268	*Veronica wormskjoldii*	Forb	BT	0	-0.18	NA	NA	1
269	*Potentilla uniflora*	Forb	BT	0	-0.12	NA	NA	1
270	*Carex nardina*	Graminoid	U	0	0.23	4.01	NA	2
271	*Arenaria pseudofrigida*	Forb	U	0	-0.91	NA	NA	1
272	*Lesquerella arctica*	Forb	U	0	-0.45	NA	NA	1

References

Daniëls, F.J.A., Gillespie, L.J. & Poulin, M. (2013). Chapter 9. Plants. In: *Arctic Biodiversity Assessment. Status and trends in Arctic biodiversity*. Conservation of Arctic Flora and Fauna, Akureyri, pp. 311–353.

Elven, R. (2007). *Checklist of the panarctic flora (PAF) vascular plants*. National Centre of Biosystematics, Natural History Museum, University of Oslo.

Meltofte, H. (2013). *Arctic Biodiversity Assessment. Status and trends in Arctic biodiversity.* Conservation of Arctic Flora and Fauna, Akureyri.

Walker, D.A., Raynolds, M.K., Daniëls, F.J.A., Einarsson, E., Elvebakk, A., Gould, W.A., *et al.* (2005). The Circumpolar Arctic vegetation map. *Journal of Vegetation Science*, 16, 267–282.
